# A patient and public involvement (PPI) toolkit for meaningful and flexible involvement in clinical trials – a work in progress

**DOI:** 10.1186/s40900-016-0029-8

**Published:** 2016-04-27

**Authors:** Heather J. Bagley, Hannah Short, Nicola L. Harman, Helen R. Hickey, Carrol L. Gamble, Kerry Woolfall, Bridget Young, Paula R. Williamson

**Affiliations:** 1 Department of Biostatistics, Clinical Trials Research Centre, Institute of Translational Medicine, University of Liverpool, Liverpool, L69 3BX UK; 2 Institute of Psychology, Health and Society, University of Liverpool, Liverpool, L69 3BX UK

**Keywords:** Research, Patient and public involvement, Toolkit, Clinical Trials

## Abstract

**Plain Language Summary:**

Funders of research are increasingly requiring researchers to involve patients and the public in their research. Patient and public involvement (PPI) in research can potentially help researchers make sure that the design of their research is relevant, that it is participant friendly and ethically sound. Using and sharing PPI resources can benefit those involved in undertaking PPI, but existing PPI resources are not used consistently and this can lead to duplication of effort. This paper describes how we are developing a toolkit to support clinical trials teams in a clinical trials unit. The toolkit will provide a key ‘off the shelf’ resource to support trial teams with limited resources, in undertaking PPI. Key activities in further developing and maintaining the toolkit are to:

● listen to the views and experience of both research teams and patient and public contributors who use the tools;

● modify the tools based on our experience of using them;

● identify the need for future tools;

● update the toolkit based on any newly identified resources that come to light;

● raise awareness of the toolkit and

● work in collaboration with others to either develop or test out PPI resources in order to reduce duplication of work in PPI.

**Abstract:**

**Background**

Patient and public involvement (PPI) in research is increasingly a funder requirement due to the potential benefits in the design of relevant, participant friendly, ethically sound research. The use and sharing of resources can benefit PPI, but available resources are not consistently used leading to duplication of effort. This paper describes a developing toolkit to support clinical trials teams to undertake effective and meaningful PPI.

**Methods**

The first phase in developing the toolkit was to describe which PPI activities should be considered in the pathway of a clinical trial and at what stage these activities should take place. This pathway was informed through review of the type and timing of PPI activities within trials coordinated by the Clinical Trials Research Centre and previously described areas of potential PPI impact in trials.

In the second phase, key websites around PPI and identification of resources opportunistically, e.g. in conversation with other trialists or social media, were used to identify resources. Tools were developed where gaps existed.

**Results**

A flowchart was developed describing PPI activities that should be considered in the clinical trial pathway and the point at which these activities should happen. Three toolkit domains were identified: planning PPI; supporting PPI; recording and evaluating PPI. Four main activities and corresponding tools were identified under the planning for PPI: developing a plan; identifying patient and public contributors; allocating appropriate costs; and managing expectations. In supporting PPI, tools were developed to review participant information sheets. These tools, which require a summary of potential trial participant characteristics and circumstances help to clarify requirements and expectations of PPI review. For recording and evaluating PPI, the planned PPI interventions should be monitored in terms of impact, and a tool to monitor public contributor experience is in development.

**Conclusions**

This toolkit provides a developing ‘off the shelf’ resource to support trial teams with limited resources in undertaking PPI. Key activities in further developing and maintaining the toolkit are to: listen to the views and experience of both research teams and public contributors using the tools, to identify the need for future tools, to modify tools based on experience of their use; to update the toolkit based on any newly identified resources that come to light; to raise awareness of the toolkit and to work in collaboration with others to both develop and test out PPI resources in order to reduce duplication of work in PPI.

**Electronic supplementary material:**

The online version of this article (doi:10.1186/s40900-016-0029-8) contains supplementary material, which is available to authorized users.

## Background

Patient and public involvement (PPI) in research is where research is “being carried out ‘with’ or ‘by’ members of the public” not just “‘to’, ‘about’ or ‘for’ them” [1]. Involving the public in research in this way is important for both moral and pragmatic reasons. Morally, PPI is advocated on the grounds that people affected by a condition, or the wider public in the case of public health research, have a right to have a say in decisions about research that may affect them. This includes how research is designed and undertaken and how research findings are disseminated and implemented once a study is complete. The slogan “nothing about us without us”, which is believed to be over five centuries old (https://en.wikipedia.org/wiki/Nothing_About_Us_Without_Us), encapsulates this argument. Pragmatically, involving the public is intended to benefit the research process by ensuring research is relevant, conducted in an appropriate ethical manner, that it is participant friendly and that the results of research are made accessible and provided with sensitivity to study participants and the wider public once the study is complete.

There are both positive and negative reports about PPI [2]. Positive impacts of PPI have been reported for various trial stages from study design and the selection of outcomes to the dissemination and implementation of findings [3–5]. Importantly, PPI may contribute to the successful delivery of trials by increasing the likelihood of recruitment to time and target together with improving participant retention [6, 7]. Potential negative impacts of PPI on research have been reported, including ‘potential scientific and ethical conflict on protocol design’, potential reduction in or biases in recruitment, power issues between researchers and patient and public contributors^1^ and premature dissemination of research findings before academic publication [2].

Challenges with PPI have been raised indicating the need for a robust evidence base [4, 8–10]. There are times when researchers need to sensitively address the challenges of PPI, for example if public contributors are perceived by the research team to have an overly narrow or self-serving agenda or are not engaging sufficiently, despite being given opportunity and support. There is evidence that PPI is inconsistent across trials in terms of engagement, quality and reporting [11, 12]. The Evidence Base for Public Involvement in Clinical Trials (EPIC) study, retrospectively examined PPI in clinical trials and UKCRC Registered Clinical Trials Units across a range of conditions [13]. The EPIC study reported variation in: planned PPI activities; in the experience of public contributors and researchers in undertaking PPI and in evaluation of reporting of the impact of such involvement. Key conclusions were that PPI works well if its goals are clear, if there are well developed plans for PPI in a trial and if models of PPI are more responsive and managerial (for example, membership of a Trial Management Group) rather than restricted to general oversight (for example, membership of a Trial Steering Committee). Evaluation of the impact of PPI in clinical trials is rarely conducted [14]. A review of the reporting of PPI in surgical research [15] indicates poor standards in PPI reporting, limiting attempts to explore the impact of PPI.

Clinical trials units (CTUs) are “specialist units which have been set up with a specific remit to design, conduct, analyse and publish clinical trials and other well-designed studies” [16]. The combined demands of running a portfolio of diverse clinical trials and intense regulatory and ethical scrutiny in CTUs demand streamlined processes that may limit flexibility and time for PPI. Indeed, the EPIC study found that within clinical trials, a key barrier to PPI implementation was time constraints [13]. Some clinical trials units have reviewed their PPI activities and identified areas for future development [17, 18]. The EPIC study surveyed UK Clinical Research Collaboration (UKCRC) registered CTUs to identify PPI practices, and reported a number of issues in implementing PPI, including problems finding public contributors for involvement; limited use of INVOLVE^2^ resources to support public contributors and a lack of evaluation of the experience of public contributors in their role [13].

In their review of barriers, drivers and impacts of public involvement in health research generally, Snape et al [19] identified that “appropriate resources were considered essential to effective PPI implementation”. This has been addressed by some CTUs through the use of standard operating procedures (SOPs) [20] with over half of UKCRC registered CTUs reporting having a SOP related to PPI in place or in development [13]. Whilst SOPs offer a structure to implement consistency in CTU procedures, these are often developed according to an individual CTU’s requirements and are likely to offer a rigid approach to PPI that is in contrast to the flexible and responsive approach recommended by Buck et al [12] based on the needs of each trial.

Although INVOLVE resources do exist to support public contributors, Gamble et al. [13] found that in a survey of CTUs only a fifth used these resources to train and support public contributors. The National Institute for Health Research (NIHR) in England also recently conducted a review of public involvement in research [8] reporting that whilst useful resources exist, such as the INVOLVE cost calculator and guidance on budgeting for involvement [21] “there is concern that this is not widely known about, and third sector representatives in particular were unfamiliar with it but could immediately see its benefits” [8]. One approach that may address this issue is the use of a well promoted, easily accessible PPI toolkit. Toolkits can be defined as “the packaging of multiple resources that codify explicit knowledge” which aim to promote knowledge sharing, education and to “facilitate behaviour change” [22]. Toolkits offer a sign-posted structure with practical, adaptable tools contained within a logically organised, user friendly, platform. Some PPI toolkits are already available e.g. within service development [23–25]. Toolkits for involving the public in NIHR Clinical Research Networks (CRNs) also exist, for example in cancer research [26]. There are many useful resources in these toolkits but they are not specific to the requirements of clinical trials units and are not designed around the aforementioned demands on such units. General guidance for clinical trials has been written [27, 28] however this does not provide the tools to support particular activities. For example guidance suggests that public contributors can help researchers with activities such as improving the quality of patient information [28], but there is a need for a tool that provides guidance to public contributors on how to review such information. The INVOLVE ‘Public involvement in clinical trials: Supplement to the briefing notes for researchers’ [27] is also available and provides general guidance and considerations for PPI in CTUs, including the benefits and challenges, along with case studies and quotations, but not specific tools for use in trials units.

This paper describes the initial phases of the development of a web based toolkit designed for use by chief investigators and study teams to facilitate meaningful and effective PPI at all stages of a clinical trial. It is hoped that dissemination of this work will facilitate the engagement of various stakeholders, including public contributors and other clinical trials units, in further development of the toolkit and in a subsequent evaluation and improvement process.

## Method

### Reviewing PPI and developing a PPI working group

In 2013, the Clinical Trials Research Centre (CTRC, http://www.liv.ac.uk/translational-medicine/research/ctrc/about/) undertook a review of their PPI activities, using a previously developed survey tool [17]. Ten trials were reviewed, the majority being paediatric trials with interventions including medicines, surgery and devices. The overall conclusion from that exercise was that there was a lack of resources and training to support the implementation of PPI within the CTRC. From that review a PPI Working Group was established within the CTRC and members include a PPI co-ordinator, the Director and Deputy Director of the CTRC, the Head of Trial Management, a senior clinical trials manager and a trial co-ordinator. The PPI co-ordinator has experience of being a public contributor in research and the Deputy Director of the CTRC was the chief investigator of the aforementioned EPIC study. Other members of the working group have experience of implementing PPI in research. The PPI Working Group did not involve any public members due to funding, time and resource constraints but we plan to engage with public contributors in further developing the toolkit.

The PPI working group meet regularly and identified the need for a toolkit to sign-post members of the research team to PPI resources and templates relevant to the various stages of the clinical trial. Areas for PPI development included planning, supporting, monitoring and documenting PPI. Within the CTRC, the Consent Working Group considers consent processes related to the CTRC portfolio, which includes paediatric and adult trials across a variety of conditions. Following discussion about the variability in approaches within the CTRC to gain PPI input into trial documents, the group developed resources (described below) that were integrated into the wider PPI toolkit. The initial development of other PPI resources was allocated to two members of the PPI working group (HB, HS) with other members commenting on drafts for the toolkit.

### Development of the toolkit

**Fig. 1:**
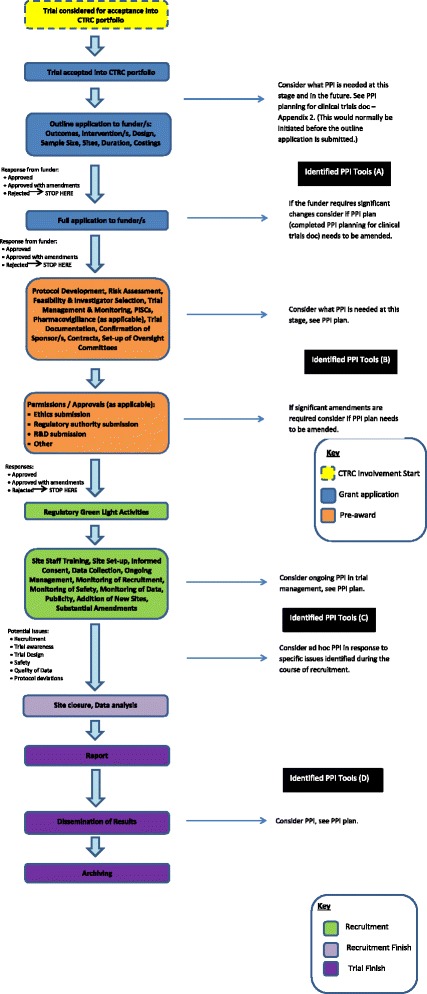
PPI in the Lifecycle of a Clinical Trial

There will be five phases to the development of our toolkit. The findings from phases 1 and 2 are presented in this paper. Phase 1 involved the description of the PPI pathway through a clinical trial. Phase 2 has involved the identification of existing resources and additional resources required (this phase is ongoing as more resources are identified or become available). Phase 3 will involve the development of a web-based resource that maps the process in Fig. 1 with links to the resources described here. Phase 4 will involve evaluating the tools included in the toolkit from the perspective of our public contributors, trial teams and the wider CTU network. Phase 5 will involve making improvements and modifications to the tools based on the experience of using them and disseminating information about the use and impact of the resources to the wider PPI in research community.

### Phase 1: Describing the PPI pathway through a clinical trial

The first phase in developing the toolkit was to describe which PPI activities should be considered in the pathway of a clinical trial and at what stage of the trial pathway these activities should take place. This pathway was informed through a review, described above, of the type and timing of PPI activities within CTRC coordinated trials. The PPI working group considered the feasibility of the pathway and whether previously described areas of potential PPI impact in trials (eg. Staley [4]) would be adequately covered through the process.

### Phase 2: Identifying existing resources and additional resources required

The identification of resources for the toolkit has involved looking at key websites around PPI as well as finding resources opportunistically, for example in conversation with others in clinical trials units or through social media. The resources identified to date were reviewed by the PPI working group and included if considered useful upon review. Organisations with PPI resources on their websites include INVOLVE, the Research Design Service (RDS), the NIHR and other organisations with very specific roles, such as around prioritising research topics (The NETSCC James Lind Alliance - http://www.lindalliance.org/) and around agreeing outcomes of importance for trials (the international COMET Initiative - http://www.comet-initiative.org/). Each identified tool was categorised under one or more of the three key toolkit domains:

- Planning PPI
- Supporting PPI
- Recording and Evaluating PPI

As other resources become available these will be identified and reviewed for potential inclusion in similar ways.

## Results

### Phase 1 Describing the PPI pathway through a trial

The PPI Working Group identified numerous activities where PPI can contribute to clinical trials:

- Defining the most relevant research question to ask within a clinical trial;
- Identifying the outcomes of importance to be measured within a clinical trial;
- Developing a clinical trial protocol appropriate to the needs and lifestyles of the patient community it serves;
- Identifying appropriate and ethically acceptable research tools and methods;
- Developing clinical trial participant materials, including but not limited to the patient information sheet and consent form, patient diaries and questionnaires;
- Conducting the trial in a participant friendly and ethically acceptable way;
- Providing a public perspective on the interpretation of trial findings;
- Disseminating the results (to both trial participants, the general public and health professionals) to ensure awareness of study findings and adoption of the trial results in clinical practice and
- Measuring the impact of a trial’s findings and informing future trial design.

The working group produced a flowchart describing the PPI activities that should be considered in the clinical trial pathway and the stages of the pathway where these activities should happen (Fig. 1).

### Phase 2 Identifying existing resources and additional resources required

Numerous PPI resources have been identified and these have either been included as links within the toolkit or have been adapted for use by the CTRC. Although we provide links we are not endorsing these resources, but are suggesting they may prove useful for researchers undertaking PPI. The toolkit is a living document and new or currently unidentified PPI resources that come to light will be added or modified as required. The toolkit sign-posts researchers to relevant PPI resources along the timeline of the trial to ensure existing tools are more visible and accessible. In developing this resource we are conscious that the tools need to go further than just providing knowledge. Figure 1 further shows the groups of identified tools for specific aspects of the trial and the tools are presented in Additional file 1: Appendix 1. We describe below the rationale for resources and tools that we have so far developed, and the activities identified under each of three toolkit domains.

#### Planning for PPI

Four main activities and corresponding tools were identified under the planning for PPI domain: (i) Developing a plan for PPI in a trial; (ii) identifying public contributors with appropriate experience and skills; (iii) allocating appropriate costs; and (iv) managing the expectations of public contributors.

I. Developing a plan for PPI in a trial
  The CTRC PPI working group acknowledged the importance of encouraging chief investigators and research teams to plan for meaningful PPI in their clinical trial and to develop plans to assess the impact of such involvement. To help achieve this, a “PPI planning tool” was developed along with guidance for this planning tool (Additional files 2 and 3: Appendix 2 and 3 respectively) which also sign-posts researchers to examples of impact assessment within the Public Involvement Impact Assessment Framework (PiiAF) resource (http://piiaf.org.uk/). This PPI planning tool includes issues for the CI and research team to consider in relation to the type of PPI input that might be appropriate, as well as planning for the assessment of impact in PPI activities. It encourages teams to question whether the opinion of one or two patients is sufficient to provide the patient perspective on an aspect of the trial or if wider consultation is needed and if so, how this could be achieved. For example, when considering which outcomes to use in a trial we wanted to challenge trial development teams to question whether asking one or two patients would provide a sufficiently inclusive perspective on which outcomes would be most important to measure in their trial. We wanted to raise awareness of other initiatives, such as the Core Outcome Measures in Effectiveness Trials (COMET) Initiative, which provides a database of studies where agreed sets of core outcomes have been defined for particular conditions and interventions, increasingly with patient involvement. Links were also provided, within the PPI planning tool, to other relevant resources.
II. Identifying public contributors
  PPI needs public contributors to be identified but this raises questions about who should be involved and the skills they may need. Identifying public contributors can be challenging [29, 30]. Existing resources were identified to support researchers in finding public contributors although they were specific to cancer patients in one resource [28] and less extensive than we planned in another [31]. To help overcome this challenge a tool was developed within the CTRC “How to find public contributors” (Additional file 4: Appendix 4). This document includes information on possible sources of public contributors, ways to advertise opportunities for involvement and a list of organisations that may provide routes to access potential contributors.  Another important aspect is to be clear about the skills and experiences that are relevant to public contributors' roles. Future tools will be developed to support research teams in the selection of public contributors, including the match between their capabilities and the needs of the CTU or trial.
III. Allocating appropriate costs
  As the PPI activities of a trial are identified, consideration needs to be given to the cost of completing these activities so that they are achievable, compensate contributors for their contributions and do not leave them out of pocket. INVOLVE and the Mental Health Research Network (MHRN) in England have developed a document “Budgeting for Involvement” [21] and a corresponding involvement cost calculator which suggests that the following costs are considered: payment and rewards, individual expenses to include travel, subsistence and childcare, training costs, costs of involvement activities including venue costs and equipment, involvement staffing including coordinators and facilitators and other costs such as translation or supporting individuals with additional needs. Whilst this resource offers guidance on what costs to consider it is up to the research team to attribute appropriate costs under each of these areas. To aid research teams and standardise costs for trials coordinated by the CTRC, the CTRC internal grant costing template includes rates for PPI items and activities based on INVOLVE guidance, the amount of time required and the mode of communication.
IV. Managing the expectations of public contributors
  Planning PPI also needs consideration of what is expected of public contributors. The CTRC have developed template PPI remit documents for involvement in the Trial Management Group (TMG) and the Trial Steering Committee (TSC, Additional files 5 and 6: Appendix 5 and 6 respectively). These documents include basic information on: the background to the trial and the relevant committee (including a flowchart of trial timelines); the role of the public contributor; membership and key contacts; frequency and format of meetings; confidentiality; honorarium payment and expenses (and implications of payment for involvement) and evaluation and monitoring of the impact of PPI.

#### Supporting PPI

There are few tools available to support specific PPI activities. Within the CTRC Consent Working Group it was noted that across trials PPI input is consistently sought for the trial participant information sheet and consent form, but the methods of seeking this input are inconsistent and may not provide suitable support and information for public contributors. To address this, two documents were developed. The first was a question bank of items for public contributors to consider when reviewing a participant information sheet (the Participant Information Review Question Bank). Relevant questions are to be selected from this bank by the trial team. The second resource is a guidance document which provides a summary of patient characteristics and circumstances for the trial and clarifies researcher requirements and expectations of PPI review, populated with questions from the question bank (this second resource is the Participant Information Review – Guidance for Public Contributors). Both documents are currently being piloted within the CTRC (Additional files 7 and 8: Appendix 7 and 8 respectively).

Other resources will continue to be developed and/or links to existing resources identified including: training documents that include an induction template outlining areas to cover as part of welcome meetings; tools to support specific activities, for example, commenting on trial design, recruitment strategies and end of trial summaries; tools to help maintain engagement of public contributors throughout the trial.

#### Recording and evaluating PPI

Whilst there is consensus that PPI has considerable potential to benefit clinical trials, there has been little formal evaluation of its impact. PPI can impact both the experience of the public contributor and research team as well as potentially influencing the trial, for example in terms of improved accessibility of participant information sheets. The PPI planning tool already described has a dual purpose - as well as helping research teams plan for PPI, the tool has been designed to also facilitate recording of the impact of the PPI intervention. In terms of evaluating the experience of PPI, evaluation tools will be developed to monitor this from the perspective of both the public contributor and the trial team. Jointly these tools will enable teams to ensure that they are adequately supporting their public contributors and will create a clear picture of when and where PPI happened, what impact this had on the trial, as well as aiding recording of PPI activities and impact for feeding back to funders.

## Discussion

The benefits and challenges of PPI in research are increasingly being recognised. The drive to undertake PPI within research is apparent with many funding bodies now requesting that researchers provide evidence of PPI when submitting their proposals including clear plans for PPI activities throughout the proposed research [32–36]. Indeed, one of the strategic goals for the NIHR is that, by 2025, PPI is a ‘required part of high quality research’ [8].

Research has shown how resources to support public contributors and researchers (INVOLVE resources) are often not used [8, 13]. The developing PPI toolkit described in this paper begins to address this problem.

### The developing toolkit

Through mapping out the clinical trial pathway (Phase 1) and exploring resources that might facilitate PPI across this pathway (Phase 2) the CTRC have identified that numerous resources currently exist to support PPI in research or that were suitable for adaptation by the CTRC. We have also identified the need for additional tools that could be developed to address current gaps in the toolkit. These will either be developed locally by the CTRC PPI working group or in collaboration with others involved in PPI in other trials units. The tools within the toolkit will be designed to facilitate meaningful involvement by promoting clear planning of PPI from the outset, by providing tools that encourage a considered approach to involvement activities, and by providing ‘off the shelf’ resources, available online, to support clinical trials, as researchers are sometimes required to develop trials at a fast pace. Phase 2 is ongoing and only preliminary findings have therefore been presented. It is anticipated that by disseminating this work while the toolkit is in development, we will facilitate the engagement of various stakeholders, including public contributors and other clinical trials units in the toolkit’s ongoing development, thereby improving its quality.

Trial teams, including public contributors, will be encouraged to provide evidence of the impact of PPI interventions against planned PPI and examples of any further resources successfully used to undertake and support PPI. It is intended that this collective ownership of the resource will promote enthusiasm for and engagement with PPI. By providing a link in the toolkit to the Guidance for Reporting Involvement of Patients and Public (GRIPP [9]) checklist on reporting of PPI we aim to support and encourage teams in publishing their PPI activities, including where trials are not funded (there may be useful examples of PPI even where a trial does not get funded). By working with other trials units we may also be able to collectively test out tools to examine to how well they support the meaningful involvement of public contributors in research. We will seek to engage trials teams in this process. This fits with a further strategic PPI goal of the UK NIHR, ensuring “evidence of what works is accessible so that others can put it into practice” [8].

One of the problems with existing resources not being used is that they are not immediately visible to all researchers and trials teams, often working on many other aspects of the trial. Within our CTRC, members of the PPI working group are working to raise awareness of the developing toolkit and resources within it. An initial training session about the toolkit was hosted at a recent Trial Managers meeting. Ongoing activity by the PPI working group will ensure that the toolkit is regularly promoted as a standing item (with information on updates to the Toolkit) at the Trial Managers meeting. Part of the induction of new trial managers will include information on PPI and the toolkit. Ideally the full trial team should discuss the tool together when planning their research and conducting their trial, but it is recognised that working practices vary. We envisage that statisticians need to be aware of the issues that patients can contribute to. Within the CTRC we expect our senior trials managers to raise awareness of the toolkit with research teams on whichever trial they are working on.

In developing our initial toolkit we recognise that a limitation of our work is that we have not involved patients and the public in its design, but due to restrictions of funding, resource and time the authors decided to develop the initial structure of the toolkit internally. Having this initial structure will enable public contributor engagement with a developing resource, rather than starting with a blank slate. The lead author is also a public contributor in research and clinical trials and this has therefore provided an initial public steer to the work.

### Next steps

There have been calls for greater collaborative working to avoid duplication of work in patient and public involvement. The recent NIHR strategic review of public involvement in research in the UK emphasised “the importance of partnership and collaboration to future success” [8]. The CTRC has worked to develop a network of PPI professionals working in UK CTUs. The CTRC sent an invitation via the UKCRC Registered CTU Network for people with a specific role in PPI in trials units to take part in a teleconference. Twenty-two people from CTUs across the UK expressed an interest including both individuals with a specific PPI role, such as PPI co-ordinators and others, for example, senior trials managers where responsibility for PPI in a CTU was part of their wider role. In April 2015, the CTRC hosted an initial teleconference with this network to explore ways of working in collaboration. A future face to face meeting is planned to further progress collaborative working which may include the development and evaluation of PPI tools, including assessment of the value of elements of the toolkit presented here. We anticipate this group may offer to liaise with their patient panels and public contributors to engage with this process of assessment. We hope that through this consensus process we will optimise the resources made available in the toolkit. This meeting will also offer an opportunity to consider how the success of the toolkit should be measured, since there is little information in the literature of relevance to this issue.

In further developing and reviewing all the tools for the toolkit, and the structure of the toolkit itself, we will involve not only CTRC public contributors and trial teams, but also interested collaborators from the wider UKCRC Registered CTU network and their public contributors, so ensuring that we have the most appropriate and user friendly tools possible and suitable active links to other relevant resources.

Although this toolkit has been designed in the UK, the stages of a clinical trial are similar internationally and therefore many of the resources in this toolkit are relevant to the implementation of PPI in clinical trials in other countries. Indeed, PPI is growing internationally. While the development of this toolkit has not involved a systematic review of resources worldwide, as part of the ongoing development of the toolkit we intend to explore PPI resources internationally. This may include: the Patient Centred Outcomes Research Institute (PCORI, USA); Involving People (Wales); Involving People in Research (Australia); Health Technology Assessment International (HTAI) and the European Patients Academy on Therapeutic Innovation (EUPATI). Where relevant resources are identified from these and other sources we will incorporate links to these.

The toolkit will be further developed and continually informed both by activities that identify gaps or resource needs and by ongoing revision of resources and links based on any newly available research, information or tools to make sure that the toolkit remains current. This work, and the online provision of these resources will be facilitated by CTRC infrastructure funding for the PPI Coordinator post and information systems support. The usefulness of the toolkit and the appropriateness of the resources will be assessed through formal evaluation of public contributor and trial team experience.

## Conclusion

Patient and public involvement in research is often surrounded by fears of tokenistic approaches [12] and although numerous resources exist to support PPI these are infrequently used [8, 13]) and are not specific to clinical trials which operate under different ‘rules’ to other areas of research. By signposting researchers to key practical resources, examples and links along the life cycle of a trial, our toolkit will ensure that trials teams are not duplicating effort in developing such documents from scratch, but instead have ‘off the peg’ tools that they can review and adapt as appropriate for their trials. It is anticipated that the resources will encourage teams to question and reflect on their plans and thereby to maximise the impact of public involvement in their research. This is similar to the philosophy of the Trials Forge initiative, which encourages the questioning of evidence for all trial decisions [37]. Working collaboratively with other trials units will also provide opportunities for testing and improving our resources, adding in additional resources as these are identified and potentially developing new resources and generating evidence of what works best in PPI for clinical trials.

### Endnotes

^1^Patients and members of the public who have PPI roles in research are often referred to as a ‘PPI representatives’. However, this implies that one or a small number of individuals can represent the perspectives of diverse patient groups and members of the public. Therefore, for the purposes of this paper we use the term ‘public contributor’.

^2^INVOLVE is a national advisory group that supports greater public involvement in NHS, public health and social care research in England.

## Additional files

Additional file 1: Appendix 1. List of Tools. (PDF 303 kb)

Additional file 2: Appendix 2. PPI Planning Tool. (PDF 202 kb)

Additional file 3: Appendix 3. PPI Planning for Clinical Trials – Guidance for Chief Investigators. (PDF 213 kb)

Additional file 4: Appendix 4. How to Find Public Contributors. (PDF 302 kb)

Additional file 5: Appendix 5. PPI Remit TMG Public Contributor. (PDF 711 kb)

Additional file 6: Appendix 6. PPI Remit TSC Public Contributor. (PDF 807 kb)

Additional file 7: Appendix 7. Participant Information Review Question Bank. (PDF 441 kb)

Additional file 8: Appendix 8. Participant Information Review - Guidance for Public Contributors. (PDF 523 kb)

## Abbreviations

COMET: Core Outcome Measures in Effectiveness Trials Initiative
CRN: Clinical Research Network
CTRC: Clinical Trials Research Centre
CTU: clinical trials unit
EPIC: Evidence Base for Public Involvement in Clinical Trials (study)
EUPATI: European Patients Academy on Therapeutic Innovation
GRIPP: Guidance for Reporting Involvement of Patients and Public (checklist)
HTAI: Health Technology Assessment International
MHRN: Mental Health Research Network
NETSCC: NIHR Evaluation, Trials and Studies Coordinating Centre
NIHR: National Institute for Health Research
PCORI: Patient Centred Outcomes Research Institute
PiiAF: Public Involvement Impact Assessment Framework
PPI: Patient and Public Involvement
SOP: Standard Operating Procedure
TMG: Trial Management Group
TSC: Trial Steering Committee
UKCRC: UK Clinical Research Collaboration
