# Supplementary material for: A patient and public involvement (PPI) toolkit for meaningful and flexible involvement in clinical trials – a work in progress
Source: Res Involv Engagem. 2016 Apr 27;2:15. doi: 10.1186/s40900-016-0029-8 (PMC5611579; doi:10.1186/s40900-016-0029-8)
Supplement: Supplementary file 1 — List of Tools. (PDF 303 kb) [file 40900_2016_29_MOESM1_ESM.pdf]

Appendix 1 – List of Tools

Tools (A)

PPI Activity	Tools	Developed / in development by CTRC	Requires development	External links
**Planning PPI**	PPI planning tool (Appendix 2)	x		
	PPI planning tool guidance (Appendix 3)	x		
	How to Find Public Contributors (Appendix4)	x		
	CTRC PPI Costing Template	x		
	INVOLVE cost calculator http://www.invo.org.uk/resource-centre/payment/involvement-cost-calculator/			x
	INVOLVE Budgeting for Involvement http://www.invo.org.uk/wp-content/uploads/2013/07/INVOLVEMHRNBudgeting09Jul2013.pdf			x
	INVOLVE Guidance on the use of Social Media http://www.invo.org.uk/posttypepublication/guidance-on-the-use-of-social-media/			x
	GRIPP checklist[8]			x
	INVOLVE Diversity and Inclusion Document			x
PPI in Trial Design	PPI in Trial Design Question Bank		x	
	Template Study overview – PPI consultation document		x	
	Template instructions for providing feedback on trial design		x	
Preparing for involvement	PPI Remit document – Trial Management Group (Appendix 5)	x		
	PPI Remit document – Trial Steering Committee (Appendix 6)	x		
	PPI Recruitment and Selection resources	x		
Training on PPI for trial teams	PPI Training Needs Analysis for Trial teams		x	
	North West People in Research Forum (NWPIRF) Training, Learning and Development Resource http://www.northwestpeopleinresearchforum.org/training/			x
	Healthtalkonline clips on PPI in			x

	Research http://www.healthtalk.org/peoples-experiences/improving-health-care/patient-and-public-involvement-research/what-patient-and-public-involvement-and-why-it-important			
	Relevant areas /activities in the Public Involvement Impact Assessment Framework (PiiAF) http://piiaf.org.uk/			x
	INVOLVE Public involvement in clinical trials: Supplement to the briefing notes for researchers http://www.nihr.ac.uk/get-involved/INVOLVEpublicinvolvementinclinicaltrialsBriefingnotes2012.pdf			x
	Patient and Public Involvement How to guide: http://www.rds-nw.nihr.ac.uk/public-involvement/how-to-guide/			x
	Links to relevant webinars E.g The COMET initiative: http://www.comet-initiative.org/resources/publicinvolvement			x
PPI Recording	PPI recording tool	x		

Tools (B)

PPI Activity	Link to PPI resources	Developed / in development	Requires development	External links
PPI Recruitment and selection	Suite of PPI recruitment and selection resources	x		
	Equality and Diversity Policy for PPI	x		
Welcoming public contributors	Welcome meeting template agenda	x		
	INVOLVE Public Information Pack including the Jargon Buster http://www.invo.org.uk/posttypepublication/the-public-information-pack-pip/			x
	Induction template and materials		x	
	TwoCan Associates for the UKCRC			x

	and NCRI (2010) Patient and public involvement (PPI) in research groups – Guidance for Chairs. http://www.crn.nihr.ac.uk/wp-content/uploads/GuidanceforCSGChairs.pdf			
PPI in Participant Information Resources	Training on producing participant information sheets and consent forms	x		
	Template participant information sheet & consent form (structure and layout)	x		
	Participant information review - Question bank (Appendix 7)	x		
	Participant information review – Guidance for Public Contributors (Appendix 8)	x		
Public contributor training resources	Information for clinical trials teams			
	INVOLVE – Developing Training and Support resource http://www.invo.org.uk/resource-centre/training-resource/			x
	Links to the NWPIRF Training database - http://www.northwestpeopleinresearchforum.org/training/			x
	Information for patients			
	Links to relevant webinars & plain language summaries eg. COMET plain language summaries http://www.comet-initiative.org/resources/PlainLanguageSummary and Cochrane – What are systematic reviews? http://cccrg.cochrane.org/what-are-systematic-reviews			x
	What to look for in a research application – A guide for lay reviewers: http://clahrc-peninsula.nihr.ac.uk/uploads/attachments/PPI/Guide-to-lay-reviewing.pdf			x
	Building Research Partnerships www.macmillan.org.uk/researchlearning			
	EUPATI Patient Advocate Toolbox (when available) &webinars			x
	Center Watch Glossary of Clinical Trials Terms - http://www.centerwatch.com/heal			x

	th-resources/glossary/			
	Critical Appraisal Skills Programme e-learning resources: http://www.casp-uk.net/#!checklists/cb36			x
Supporting PPI	CTRC Process for Making PPI Payments Resource	x		
	INVOLVE (2010) Payment for involvement: A guide for making payments to members of the public actively involved in NHS, public health and social care research (resource currently being updated): http://www.invo.org.uk/posttypepublication/payment-for-involvement/			x
	INVOLVE What you need to know about payment (for public contributors) http://www.invo.org.uk/posttypepublication/what-you-need-to-know-about-payment/			x
	Top Tips for Maintaining PPI Interest in a Trial Resource		x	
	Guidance on providing feedback and ongoing acknowledgement and support to public contributors		x	
	Public contributor into trials newsletter		x	
	Template PPI evaluation and impact document		x	
	Instructions on use of the evaluation and impact document		x	
	Dealing with difficulties with PPI		x	

Tools (C)

PPI Activity	Link to PPI resources	Developed / in development	Requires development	External links
Maintaining PPI interest in a Trial	Top Tips for Maintaining PPI Interest in a Trial Resource		x	
	Guidance on providing feedback and ongoing acknowledgement and support to public contributors		x	
	Public contributor newsletter		x	
Evaluating PPI Experience	Template evaluation document	x		

Resources				
	Process of disseminating / returning / responding to evaluation forms		x	

Tools (D)

PPI Activity	Link to PPI resources	Developed / in development	Requires development	External links
PPI into Written Study Dissemination Resources	Return of Results Guidance Document and Toolkit: http://mrctcenter.org/return-results			x
	Template End of Study participant information and Public Information on Study Results (for structure and layout		x	
	Question bank for consulting over End of Study Participant Information and Public Information on Study Results – examples of questions to ask public contributors when asking for comments on these two documents		x	
	Template PPI Feedback Reviewer Information Sheet		x	
	Critical Appraisal Skills Programme (CASPP guidance for public contributors			x
Dissemination of PPI Activities	GRIPP checklist[8] checklist for reporting on PPI activities			x
Public Contributor Presenter Resource	Guidance on preparing a Public Contributor for a role in presenting study results		x	
Ending PPI Resource	Top tips on ending PPI well		x	
