# Supplementary material for: A patient and public involvement (PPI) toolkit for meaningful and flexible involvement in clinical trials – a work in progress
Source: Res Involv Engagem. 2016 Apr 27;2:15. doi: 10.1186/s40900-016-0029-8 (PMC5611579; doi:10.1186/s40900-016-0029-8)
Supplement: Supplementary file 2 — PPI Planning Tool. (PDF 202 kb) [file 40900_2016_29_MOESM2_ESM.pdf]

Patient and Public Involvement Planning Tool

Topic	Questions To Consider	Has this question been addressed?			Planning for PPI & assessing its impact						
					Which PPI options are needed to answer this question						
		Yes		No							
		When? (Pre-Award / Post-Award / Pre and Post Award)	Provide details including dates and type of PPI involvement and impact	If no provide reason	Public contributor/s on Trial Management Group	How will you assess the impact of public involvement?	Consultation with small group of public contributors	How will you assess the impact of public involvement?	Consultation / survey with larger group of public contributors	How will you assess the impact of public involvement?	Other, specify
	Is this research question a priority for patients?										
Relevance	Has this topic / research question been prioritised by an existing project that had patient involvement e.g. James Lind Alliance Priority Setting Partnership: http://www.lindalliance.org/top-tens.asp?										
	Does a core outcome set exist for this condition? (Consider whether the core outcome set was developed with patients / carers and how many).(e.g. http://www.comet-initiative.org/)										
Outcomes	Do you know (from the expertise of a patient perspective) whether the outcomes intended for use in the study are relevant for patients (those outcomes that you intend to use in addition to the core outcome set, if one exists, or all outcomes if it doesn't)?										
	If you are developing patient questionnaires specifically for this trial do you know (from the expertise of a patient perspective) whether the questionnaires are relevant, appropriate and understandable? (E.g. PROMIS - http://www.nihpromis.org/?AspxAutoDetectCookieSupport=1#2)										
	Do you know (from the expertise of a patient perspective) what ethical issues there may be with the trial?										
Ethics	If there are potentially complex ethical issues with the trial, has qualitative research already been conducted with patients with regards your approach to consent e.g. Deferred consent.										
	If there are potentially complex ethical issues with the trial, do you need to gain additional patient perspectives about the aforementioned complex ethical issues in your trial?										
	Has previous research been conducted with patients about specific methodological aspects of your trial?										
	Do you need to gain further patient perspectives on methodological issues relevant to your trial?										
	Do you know from the expertise of a patient perspective:										
	· What potential patient preference issues there may be with your trial?										
	· What concerns patients might have with the risks identified in this trial?										

Topic	Questions To Consider	Has this question been addressed?			Which PPI options are needed to answer this question						
		Yes		No							
		When? (Pre-Award / Post-Award / Pre and Post Award)	Provide details including dates and type of PPI involvement and impact	If no provide reason	Public contributor/s on Trial Management Group	How will you assess the impact of public involvement?	Consultation with small group of public contributors	How will you assess the impact of public involvement?	Consultation / survey with larger group of public contributors	How will you assess the impact of public involvement?	Other, specify
Logistics	· What concerns patients might have with the likely burden of your trial on a patient / family?										
	· What practical issues patients might be concerned about with regards your trial (e.g. frequency / timing/ location of visits / interventions / examinations)?										
	· What concerns patients may have with the planned investigations / interventions in the trial and the risks associated with these?										
	· Whether there are specific issues that may be of concern for particular patient groups in your trial e.g. patients with additional learning needs / patients with specific beliefs / patients from co-morbidities / patients without transport etc.										
	· How best to raise awareness about your trial to the appropriate patient group?										
	· How best to maintain patient interest in this trial over time?										
	· How to address recruitment challenges from a patient perspective during the trial?										
	Do you know (from the expertise of a patient perspective):										
	· How readable and understandable your plain language summary is?										
	· How readable and understandable your patient information and consent forms are?										
	· How best to address any required changes to patient information sheets / consent forms during the trial to ensure that the changes are readable and understandable to patients?										
	· What information patients might like to receive through your website / newsletters?										
	· How navigable / useful and understandable your patient information is on your website (at the outset and during your trial)?										
	· How readable and understandable your newsletters are (if these are to be used in your trial)?										
	· How patients will want to be provided access to the study results at the end of the trial?										

Topic	Questions To Consider	Has this question been addressed?			Which PPI options are needed to answer this question						
		Yes		No							
		When? (Pre-Award / Post-Award / Pre and Post Award)	Provide details including dates and type of PPI involvement and impact	If no provide reason	Public contributor/s on Trial Management Group	How will you assess the impact of public involvement?	Consultation with small group of public contributors	How will you assess the impact of public involvement?	Consultation / survey with larger group of public contributors	How will you assess the impact of public involvement?	Other, specify
Information and dissemination	· How readable and understandable your end of study participant information sheets are?										
	· The best ways of disseminating your study results to the wider patient community at the end of your trial?										
	· How readable and / or understandable your information about the study results is for the wider public (either as written information or as a presentation to patient groups)?										
Trial results	Is your interpretation of the trial results / sub study results, the same as patients / members of the public might have?										

	Documenting PPI activities and impact							
	**Which PPI options have been used to answer this question**							
How will you assess the impact of public involvement?	Public contributor/s on Trial Management Group	Impact of public involvement activity	Consultation with small group of public contributors	Impact of public involvement activity	Consultation / survey with larger group of public contributors	Impact of public involvement activity	Other, specify	Impact of public involvement activity

	Which PPI options have been used to answer this question							
How will you assess the impact of public involvement?	Public contributor/s on Trial Management Group	Impact of public involvement activity	Consultation with small group of public contributors	Impact of public involvement activity	Consultation / survey with larger group of public contributors	Impact of public involvement activity	Other, specify	Impact of public involvement activity

	Which PPI options have been used to answer this question							
How will you assess the impact of public involvement?	Public contributor/s on Trial Management Group	Impact of public involvement activity	Consultation with small group of public contributors	Impact of public involvement activity	Consultation / survey with larger group of public contributors	Impact of public involvement activity	Other, specify	Impact of public involvement activity
