# Supplementary material for: A patient and public involvement (PPI) toolkit for meaningful and flexible involvement in clinical trials – a work in progress
Source: Res Involv Engagem. 2016 Apr 27;2:15. doi: 10.1186/s40900-016-0029-8 (PMC5611579; doi:10.1186/s40900-016-0029-8)
Supplement: Supplementary file 3 — PPI Planning for Clinical Trials – Guidance for Chief Investigators. (PDF 213 kb) [file 40900_2016_29_MOESM3_ESM.pdf]

Patient and Public Involvement Planning for Clinical Trials – Guidance for Chief Investigators

In planning your approach to patient and public involvement (PPI) in your trial (both pre and post award) you will need to undertake the following key activities:

1. Consider what PPI is needed at each stage of your clinical trial
2. Identify potential sources of patient and public involvement
3. Consider patient and public involvement in the trial steering committee
4. Identify how you will assess the impact of public involvement activities

1. Planning PPI for each stage of the trial

At every stage of a clinical trial there is a potential need for PPI so it is helpful to consider what PPI is needed for your trial right from the start.

The PPI planning tool contains a list of common questions to be considered when planning PPI for your trial.

It should be completed at the pre-award stage i.e. before funding for the trial has been awarded by the Chief Investigator/s in conjunction with the Trial Co-ordinator.

Once the spread sheet has been completed you may identify some questions relating to PPI that have not yet been considered for your trial. If that occurs, you will need to consider if the questions need to be addressed at the pre-award stage and if so, how.

When confirmation of trial funding is received (post-award), the spread sheet should be re-visited by the Chief Investigator/s and Trial Co-ordinator to ensure that any relevant questions that could not be / were not addressed at the pre-award stage are addressed post-award.

The list of questions on the PPI planning tool is not intended to be an exhaustive list, rather a guide to common areas of PPI across clinical trials. The intention is that the list of questions can be added to as needed and that the completed spread sheet will provide a summary of all planned pre and post award PPI for your trial.

Please note; details of any unanticipated issues requiring PPI that arise post-award will be recorded on the "Log of PPI Activity" to be reviewed by the trial management group as necessary.

2. Sourcing PPI - Points to consider:

Is there a relevant patient organisation? (Use the "How to find public contributors" tool as a reference)

- If a patient organisation exists:
  - In what ways do they communicate with their patient / carer members (newsletters / e-newsletters / Facebook / Twitter followers)?

Pilot

Pilot

- If the patient organisation has to consult with its patient / carer members about a topic – how do they do that? What are their most successful methods
- Does the patient organisation have any smaller groups that have patient representation on them that might be suitable to consult with – e.g. to look at your patient information? (Some patient organisations have existing PPI groups so it is worth asking about this too).
- After discussing PPI with the patient organisation – do they appear interested / able / willing to provide PPI for this trial?
- From your discussions with the organisation, if they seem keen to help, try to find out what kind of PPI they might be able to provide e.g. public contributors for oversight groups, focus group, a larger group to respond to surveys?

- If a patient organisation does not exist, work through the other suggestions in the "How to find public contributors " tool to see if there might be other groups that could get involved e.g. there might have been a PPI group set up for a previous trial that has now finished who might be interested in helping out.

3. Trial Oversight – Trial Steering Committee

The PPI planning tool focuses on tasks which may require substantial PPI input. Whilst PPI representation is required on the Trial Steering Committee the role of the TSC is to provide trial oversight rather than to develop the trial application, protocol or associated documents.

When planning a grant you should ensure that PPI representation on the TSC is considered and costed appropriately.

4. Assessing and documenting the impact of public involvement activities

As part of planning your public involvement activities it is important to consider how you will assess the impact of your public involvement activities. Columns are set aside in the PPI planning tool to help you record your plans for this and to enable you to document the impacts. A range of resources to assist in planning impact assessment are available on the Public Involvement Impact Assessment Framework (PiiAF) website (http://piiaf.org.uk/) including examples of how others have assessed impact in research, including clinical trials. The PIiAf 'Database – Methods and tools to assess impacts: Signposting resource to published case examples of methods and tools' is available here: http://piiaf.org.uk/documents/impacts-database.pdf

Impacts can be assessed in relation to:

- Impact on the research and
- Impact on those involved in PPI, such as public contributors on a PPI panel.
