# Supplementary material for: A patient and public involvement (PPI) toolkit for meaningful and flexible involvement in clinical trials – a work in progress
Source: Res Involv Engagem. 2016 Apr 27;2:15. doi: 10.1186/s40900-016-0029-8 (PMC5611579; doi:10.1186/s40900-016-0029-8)
Supplement: Supplementary file 4 — How to Find Public Contributors. (PDF 302 kb) [file 40900_2016_29_MOESM4_ESM.pdf]

CTRC
Clinical Trials Research Centre

How to find public contributors for public involvement in trials

There are a number of options available when trying to identify public contributors to be involved in a clinical trial and these are influenced by whether or not a patient organisation exists. There are pros and cons to the different ways of finding and approaching public contributors. How you find people will depend on issues such as the aims of your project, the topic area and the needs of the people you wish to work with .

Patient Organisations

Contact a charity / patient group relevant to the condition – resources for finding such groups include:

- **Contact a Family** - www.cafamily.org.uk/medical-information/conditions - select the condition from the A-Z link – it gives you both a plain English description of the condition first and then if you click on the sub-section entitled "Is there support?" – there is a list of relevant support groups / patient organisations.
- **Patient.co.uk** - www.patient.co.uk/directory - You can search by condition and geographical area and it provides both local and national information on patient organisations / support groups.
- **Self help UK** - www.self-help.org.uk/directory - Also provide a searchable directory but you can't currently search by geographical location.
- **Rare diseases UK** - www.raredisease.org.uk/members.htm#patient – Has an A-Z list of patient organisations in rare diseases.

You can also find patient groups through social media but should be cautious as not all groups are closed groups and it may not be clear how people came to join the group. A public contributor selection process enables you to ask questions about experience of the condition which may help. INVOLVE Guidance on the use of social media for involvement is available here: http://www.invo.org.uk/wp-content/uploads/2014/11/9982-Social-Media-Guide-WEB.pdf

Patient and Public Involvement (PPI) Organisations

A number of PPI organisations can help:

- **INVO-DIRECT** - This online resource, developed and maintained by INVOLVE, is a directory of networks, groups and organisations that support active public involvement in NHS, public health and social care research. There may be relevant groups worth contacting in their directory: http://www.invo.org.uk/find-out-more/invodirect/
- The mental health research charity **McPin** has a directory of mental health service user and carer research groups across the UK: http://mcpin.org/resources/service-user-and-carer-groups/
- The following PPI in Research organisations will all promote PPI opportunities free of charge:

◊ **People in Research** - http://www.peopleinresearch.org/

- ◊ **North West People in Research Forum** - http://www.northwestpeopleinresearchforum.org/
- ◊ **Involving London** - http://www.involvinglondon.co.uk/
- ◊ **Patients Active in Research** - http://patientsactiveinresearch.org.uk/
- ◊ **Making Research Better** - https://www.makingresearchbetter.co.uk/

- **Involving People** - Researchers working in Wales can advertise opportunities with the involvement organisation Involving People - http://www.wales.nhs.uk/sites3/home.cfm?orgid=1023
- The NIHR CRN Children has a national **Young People's Advisory Group** who comment on research - the consumer Liaison manager Jenny Preston can be contacted here: Jennifer.Preston@liverpool.ac.uk
- **The Young People's Mental Health Advisory Group** - this national group are available to support researchers wanting younger people's involvement in research: https://docs.google.com/viewer?a=v&pid=sites&srcid=bmloci5hYy51a3xjcm5jZW50cmFsfGd4OjMzYTY1MjJmNTJmODcyYzg

Other Research Organisations and Networks

- **Contact the PPI lead of a relevant research Network** – they may have public contributors with an interest in your topic. For contact details for Research Network PPI Leads see:

  www.crn.nihr.ac.uk/can-help/funders-academics/our-ppi-offering-to-the-research-community

- **Contact other Research Design Services (RDS) or Collaboration for Leadership in Health Research and Care (CLAHRCs)** – These research organisations might have patients / carers / parents with relevant experience to comment. For example the CLAHRC at South West Peninsula has an active PenPIG PPI group: http://clahrc-peninsula.nihr.ac.uk/meet-the-penpigs.php. For a list of CLAHRCS see: http://www.clahrcpp.co.uk/#!clahrcs/cjg9.

NHS and healthcare education

- **Acute Trusts** - These may have patient involvement groups or public membership groups that might be prepared to promote opportunities for you
- **Local patient / public community groups** - There may be other local patient / public community groups that may be prepared to get involved, for example a local Primary Care Patient Participation Group - http://www.napp.org.uk/. (The "Research Engagement Award" was established in 2014 to recognise the good work that Patient Participation Groups (PPGs) do in primary care settings to promote research). Alternatively you could search the internet for community group directories – many local authorities hold such information on their websites.
- **Patient experience groups** - There may be patient experience groups within healthcare provider organisations who may have interested patients (eg. http://www.ipswichhospital.nhs.uk/getinvolved/the-patient-experience-group.htm; (accessed 25/11/13) http://www.kmpt.nhs.uk/Patient-Experience-Group.htm) (accessed 25/11/13) or public members of Trusts. The organisers of such groups may be prepared to promote the opportunities for you.
- **Patient involvement in healthcare education** - Organisers of patient involvement activities in medical / nursing / allied health professionals educational programmes may be prepared to promote opportunities.

- **Patient Advice and Liaison Service (PALS) teams -** Contact your local PALS team to see if they have any suitable links or networks.
- **Outpatient clinics** - Flyers could be put up in relevant clinics (including primary, secondary and tertiary care centres)

Parent Forums

- **Parent Carer Voice Groups**—There are some very active Parent Carer Voice Groups who have links with other local groups for children with additional needs eg. http://cepcvoice.org/index.html and http://www.devonparentcarersvoice.org/ . Many parents associated with these organisations are familiar with being asked to take part in consultations.
- **The National Parent Partnership Network** - This network may be worth approaching if the condition relates to children with additional needs as they have links to local support groups in their areas.

Local Patients known to the Research Team

- **Consider approaching patients known to the research team** (*Note of caution - this can sometimes prove challenging for the patient and clinician involved potentially impacting on their patient / clinician relationship and the patient may feel that they have to support the clinician's views).

Other sources

- **Participants from previous PPI discussion groups** - If you carry out discussion groups about research projects with patients and the public you could discuss potential public contributor opportunities at the end of your PPI discussion group maybe with flyers to hand out. (NOTE—If they have taken part in a discussion group on the design of the trial that you want public contributors for, they will not be independent and so it would only be appropriate to mention opportunities for a Trial Management Group **not** Trial Steering Committee)
- **The Royal Colleges** - Organisations such as the Royal Colleges may have PPI groups and they may be prepared to approach members of their groups on behalf of the trial (eg. www.rcplondon.ac.uk/what-we-do/patient-involvement/patient-and-carer-network; www.rcseng.ac.uk/patients/patient-and-public-involvment; (accessed 25/11/13)
- **Clinical Trials Units** - Other trials units with strong PPI may be prepared to promote PPI opportunities. For information on other Clinical Trials Units see: http://www.ukcrc-ctu.org.uk/
- **Non health related community groups.** E.g. engaging with schools, youth clubs, faith groups etc.

The Patient and Public Involvement Working Group at the Liverpool Clinical Trials Research Centre are keen to hear about other sources of PPI. If you know of any please contact Heather Bagley, Patient and Public Involvement Coordinator: heather.bagley@liv.ac.uk. Equally if you have any suggestions on improvements to this tool please contact Heather.

Note: We recognise that organisations are constantly changing and evolving and this tool will therefore be regularly updated
