# Supplementary material for: A patient and public involvement (PPI) toolkit for meaningful and flexible involvement in clinical trials – a work in progress
Source: Res Involv Engagem. 2016 Apr 27;2:15. doi: 10.1186/s40900-016-0029-8 (PMC5611579; doi:10.1186/s40900-016-0029-8)
Supplement: Supplementary file 5 — PPI Remit TMG Public Contributor. (PDF 711 kb) [file 40900_2016_29_MOESM5_ESM.pdf]

<STUDY ACRONYM/LOGO>

<Study full name>

Trial Management Group (TMG) Public contributor -

Remit & role description

This document outlines the background to both the <insert trial name> and the Trial Management Group and identifies the specific role and remit of patients, parents and carers who are members of this group.

Information at a glance	
Your Role	Public contributor of the <trial name> Trial Management Group
Trial Duration	<insert trial start and end dates>
Duration of public contributor role	<Insert anticipated timescale of involvement required>
Frequency of involvement	<Insert anticipated frequency of involvement>
Location of involvement	Meetings will take place via teleconference <or describe alternative if meetings face to face, skype etc>
Expenses	<delete this row / amend as appropriate based on PPI funding in the trial> An honorarium will be paid for meeting attendance via teleconference
Key contact information	<Name><br><br><telephone number><br><br><email address><br><br><address>

Contents **Page**

Background to the trial <#>

Background to the TMG <#>

Terms <#>

Public contributor qualities <#>

The role of the public contributor <#>

Membership and key contacts <#>

Frequency and format of meetings <#>

Confidentiality <#>

Evaluation and monitoring of the impact of patient and public involvement <#>

Honorarium payment and expenses <#>

Appendix 1 - Examples of possible public contributor activities <#>

Appendix 2 – Clinical Trial Timelines <#>

UNIVERSITY OF LIVERPOOL

Study Logo

<Study name in full>

What is the <Trial name> about?

<insert a very brief overview of the study, including who the trial is for and why it is being done (in plain English)>.

What is Patient and Public Involvement?

Public and Patient Involvement (PPI) and public contribution are often used interchangeably. In this document we use public contribution to refer to all such activities. In this context a public contributor refers to:

- *Children, with experience of, or interest in clinical research, health conditions and/or health settings who are interested in helping researchers design and run clinical trials*
- *Parents/carers/patients with experience of, or interest in clinical research, health conditions and/or health settings who are interested in helping researchers design and run clinical trials*

<delete depending on the trial>

What are trial oversight committees?

There are three groups that oversee the running of the trial (sometimes you will hear these three groups referred to collectively as the Trial Oversight Committees). These groups are:

- the Trial Management Group (TMG);
- the Trial Steering Committee (TSC) and
- the Independent Data and Safety Monitoring Committee (IDSMC).

The TMG, ISDMC and TSC have different roles but report to one another:

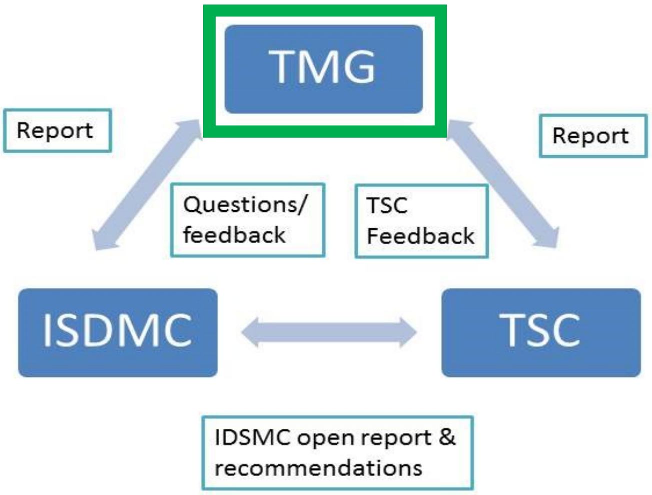

Both the TMG and the TSC usually have one or more members who are public contributors. It is important to involve people with experience of the condition to ensure the trial activities are considered from a patient perspective.

The qualities of a public contributor are in appendix 1.

Study Logo

<Study name in full>

What does the Trial Management Group do?

The TMG oversees the day to day running of the clinical trial, including issues such as recruitment, promotion and monitoring of the trial (refer to the attached <insert trial name> TMG terms of reference for full details of the role of this group).

The monitoring activities of the Trial Management Group aim to make sure that the rights and safety of the participants in the trial are protected and that the data collected is of excellent quality.

Monitoring activities of the TMG include:

- Making sure that the trial protocol is followed.
- Making sure that all approvals needed are in place, for example, from the ethics committee or hospital doing the trial.
- Looking at the planned progress of the trial and thinking about how to manage any delays.
- Making sure that the procedures in the trial meet the guidance set out for carrying out a clinical trial – referred to as Good Clinical Practice (GCP)
- Making sure that the trial is carried out according to the regulations set out in the Medicines for Human Use (Clinical Trials) Regulations 2004 <for CTIMP studies only>

What does the Independent Data and Safety Monitoring Committee do?

The IDSMC is responsible for safeguarding the interests of <trial name> trial participants and for assessing both how safe and how effective the treatments being studied are. The IDSMC also review overall progress and conduct of the trial.

The members of the IDSMC are independent and not involved in the trial in any other way. Whilst the trial is running the IDSMC is the only committee that will see the trial data presented by treatment group. The TMG and TSC will only see data for the trial participants as a whole, not for individual treatment groups.

What does the Trial Steering Committee do?

The TSC is the executive body for the trial that has overall oversight of trial activities. Membership of the TSC includes both independent members and some members of the trial team.

The TSC takes responsibility for:

- monitoring the progress of the trial
- reviewing relevant information from other sources
- considering recommendations from the Independent Data and Safety Monitoring Committee (IDSMC)
- advising the management group on all aspects of the trial for example the need for a major change to the protocol.

Study Logo

<Study name in full>

What is my role as Public Contributor member of the TMG?

The TMG is a multi-disciplinary team and each member, including public contributors, brings a different perspective, experience and knowledge to the trial team. Along with other members of the TMG you share the general role outlined in the TMG Terms of Reference (attached). As a public contributor we ask you to:

- Attend <amend as appropriate monthly> TMG meetings (these are usually by teleconference) for the duration of the trial *
- Provide a public perspective on TMG discussions
- Respond to written or verbal communications about the trial between meetings providing a public perspective
- Maintain the confidentiality of discussions and information relating to the TMG meetings and activities in accordance with the TMG confidentiality agreement (This is part of the TMG Terms of Reference).
- Report to the TMG any studies or information known to you which highlights patient related issues that may inform the work of the group.

**The level of public contributor involvement will naturally fluctuate during the trial. There will be times, particularly at the beginning following protocol development when we are setting the trial up, where we may need less input. (For further information about the stages involved in a trial see Appendix 3).*

How many meetings will I need to go to?

The frequency of the TMG meetings will be agreed by the TMG and will depend on the demands of the different stages of the trial, for example the TMG may meet monthly to begin with and then meet only every three months when the trial set up is completed. Meetings typically last for about 1 ½ hours. An online “Doodle poll” is usually set up to agree meeting dates and times.

The <Trial name> Trial Coordinator will provide you with the TMG meeting dates, times and whether they will be a face to face meeting or by teleconference.

The Trial Coordinator will also will let you know if the meetings are expected to be largely administrative. Your attendance would not be required at such meetings, but you are always welcome to attend if you want to. At quieter times, when you are not attending meetings, you can always contact the trial coordinator for an update on progress.

The TMG meetings are planned so that the main issues of relevance to public contributors are the first items on the agenda, just after the minutes from the previous meeting. This way you have the option of whether to stay for the full duration of the meeting or to leave after the items requiring public contributor input have been discussed. See appendix 2 for some of the areas of consumer involvement that you might get involved with.

Study Logo

<Study name in full>

What happens if I can't come to a meeting?

If you can't come to a meeting we would particularly value your input by email before the meeting if possible.

This might be providing comments on a document or a more general email outlining anything that you think might be important for the trial coordinator to raise on your behalf.

Do I need to do anything in between meetings?

You may be asked to comment on documents by email between meetings.

If you hear of new information/topics relating to the trial that come up between meetings, please inform your trial co-ordinator so these can be added to the TMG meeting agenda . We welcome your interest throughout the trial.

Confidentiality

All members of the TMG must abide by the confidentiality agreement set out in the <insert trial name> TMG Terms of Reference.

Who else is a member of the TMG?

The TMG is a multidisciplinary group made up of health care professionals, trial management and statistical specialists, public contributors, sponsor representatives, and other specialists who have expertise in clinical trial investigations.

- The members of the TMG are detailed in the attached <trial acronym> committee membership document
- Your main point of contact for the trial will be the Trial Co-ordinator:

<Insert name>. <phone number>
<email address>, <trial website address>

Will I receive any payment or expenses?

<Delete / amend this section as appropriate based on funding within the grant, example text is provided>

Patient/public members of the TMG will be offered an honorarium payment for preparation, attendance and participation in the Trial Management Group. This will be based on current NIHR guidance.

and/or

You will receive reasonable travel expenses for attending face to face meetings.

You can find out what you can claim for on the next page. There are some things about receiving payment that you need to be aware of and we have summarised these in appendix 4.

Study Logo

<Study name in full>

What can I claim for? *amend this section as based on what PPI has been costed for, delete section if not appropriate.*

Public contributors are entitled to claim the following expenses for each agreed training course / conference / face to face meeting they attend as a member of the TMG.

Travel to attend <delete as appropriate - face to face meetings / training / teleconferences>: (Remove this entire section if no funding for travel costs)
Taxi, bus, train and underground fares and other incidentals will be reimbursed; taxis should only be used if cheaper transport is not practicable.

- Only standard /economy class transport costs can be claimed for.
- Attempts should be made when travelling to find the most economical route possible; this particularly applies to the purchase of rail tickets and air fares. Only standard / economy class rail and air travel can be claimed for.
- Car mileage is reimbursed at a rate of 45p per mile (up to the cost of a standard class rail ticket for the same journey).

Accommodation: <delete as appropriate in line with what has been costed for in terms of any accommodation costs, remove entire section if no costs for accommodation> will be offered when travelling for trial business involves <add in conditions of accommodation for example, length or journey, start time etc that will contribute to the need for accommodation to be booked>. Accommodation should be booked via the trial coordinator prior to travel.

Childcare / carers: <Remove this entire section if no funding for registered childcare / carers> All reasonable childcare / carer costs (with registered providers) will be covered up to <insert the details based on what has been costed for >

Subsistence: <delete as appropriate in line with what has been costed for in terms of attendance at any training / conferences / teleconferences>. (Remove this entire section if no costs allocated for subsistence in the trial). With receipts:

- Whilst travelling on < insert trial name> business involving an overnight stay or where travelling results in returning home after 8.00pm, employees may claim the cost of meals. For the avoidance of doubt we will not reimburse the cost of alcoholic drinks purchased while travelling on <insert trial name> business".
- FAQ -"Can I claim subsistence whilst away on <insert trial name> business but which does not include an overnight stay? YES, lunch (reasonable) including a non-alcoholic drink can be claimed, but not dinner unless after 8pm. Does not include snacks/unlimited teas/coffees/soft drinks throughout the day.

Study Logo

<Study name in full>

What you can claim for at a glance <Delete rows and columns as appropriate depending on PPI budget>

Meeting Type	Payment	Additional expenses
Attending a face to face meeting	<Insert amount e.g. £75 per meeting>	Cost of travel<br>Childcare/carers<br>(see section 10.4)
Attending a meeting by teleconference	<Insert amount eg. £40 per meeting>	Childcare/carers
Attending a conference / training course as an observer * <insert as appropriate based on amount costed for - maximum of … conferences / training courses during the trial>	<insert amount eg. £30 per day>	The cost of travel, accommodation, subsistence and Childcare/carers<br>will be covered by the trial (see section 10.3)
Attending a conference as a presenter* <insert as appropriate based on amount costed for - maximum of … conferences during the trial>	<insert amount eg. £150 per full day>	The cost of travel, accommodation subsistence and Childcare/carers will be covered by the trial (see section 10.3)

*These activities must be agreed by the TMG / PPI co-ordinator first

How can I make a claim for expenses? *amend /delete this section as based on what PPI has been costed for*

Travel can be booked in advance by the trial coordinator and is the preferred option that also means that you are not out of pocket.

If you find that you are no longer able to attend, where possible we would appreciate it if you could provide us with 24 hours' notice so that we can cancel any transport / accommodation (although we obviously understand that sometimes emergencies happen at the last minute and this is unavoidable).

All claims for travel, childcare and subsistence expenses must be supported by receipts. <delete as appropriate to what is funded in the trial>

A claim form and further information about what can be claimed and how to claim will be provided by the Trial Manager. Alternatively you can download a copy here: https://www.liv.ac.uk/intranet/media/intranet/humanresources/2014/myhr/formstore/payroll/Non-Staff,Expense,Form.pdf

The teleconference calls will be via a Freephone number when called from a landline number. These calls should also be free in the UK when called from a mobile (as of June 2015) but please check with your mobile 'phone provider.

Will I have to do anything else in my role as public contributor?

In addition to your role in the TMG we would also like you to be involved as a public contributor in the wider context of CTRC. This would involve:

- Completing public contributor evaluation forms about your experience .
- Ensuring any comments that you make in meetings that are taken on board for the trial are documented at each meeting (in both the minutes and the evaluation form).
- Contributing to consultation exercises carried out by CTRC in relation to public contributors (for example, we might want to see what you think about the general design of our patient information sheets).

Evaluation and monitoring of the impact of patient and public involvement

It is important for us, as coordinators of the trial, to evaluate the things that we do so that we can make changes and improve them.

After each TMG meeting we will ask you to complete an evaluation form.

Completing the evaluation form is voluntary but we would greatly value your feedback so that we can make sure we are giving you the right support.

We have tested the form and it should take you about 5 minutes to complete.

The main things we want to find out are:

- How you are enjoying your involvement
- What is being done well to involve you in the TMG
- What could be done better by the TMG
- Any suggestions you have
- Any impact that you feel you have had as a public contributor either in the meeting or through correspondence between meetings.

Study Logo

<Study name in full>

Who can I contact for more information?

If you would like more general information about being a public contributor on a research study please contact Heather Bagley (CTRC Patient and Public Involvement Co-ordinator – heather.bagley@liverpool.ac.uk).
Please note Heather works part-time and term-time only. Her working days are usually Monday – Thursday 9 – 2.30 although this occasionally varies

For information about the <trial name> contact:
<trial coordinator name, telephone number and email>

The National Institute for Health Research (NIHR) funds an organisation called INVOLVE which promotes active public participation in NHS, public health and social care research to improve the way that research is prioritised, commissioned, undertaken, communicated and used. INVOLVE have a range of resources which may be of value to public contributors - www.invo.org.uk

Other information you might find helpful

Appendix 1
Qualities of a public contributor

Appendix 2
Examples of possible public contributor activities

Appendix 3
Clinical Trial Timeline

Appendix 4
The implications of being paid for involvement

Study Logo

<Study name in full>

Appendix 1—Qualities of a public contributor

<Add or delete criteria as appropriate – for every project or panel there will be different criteria and needs>

Public contributors should have experience and knowledge of <insert the name of the condition>

- As a patient, or
- As a family member or carer of a patient

Essential criteria

- Understanding of the issues relating to having <insert name of condition>
- Be able to maintain confidentiality (keeping information about the trial confidential, not discussing any information about the trial outside of the TMG)
- Ability to work effectively in a group situation
- Good communication skills with an ability to listen to others and constructively express a lay view beyond their own personal experience
- Have the time to attend meetings via teleconference / face to face meetings <delete as appropriate for your trial, depending upon whether PPI was budgeted via teleconference or whether travel for face to face meetings was budgeted in> Have the time to comment on written information / emails between meetings

Desirable criteria

- Understanding of clinical trials or experience of taking part in a clinical trial
- Access to a computer, email and telephone <This section may need adapting for certain populations where access to a computer and the internet could be a problem>

Study Logo

<Study name in full>

Appendix 2—Examples of possible public contributor activities

There are specific skills and experience that a public member of a TMG brings. Your job is to bring the patient perspective to discussions with the TMG. Listed below are examples of activities you might want to take part in , but each individual is different and so the types of activities consumers take part in vary from person to person. Activities might include:

The patient information sheet and consent form.

As someone who has used <insert the condition under investigation> services (either as a patient or a carer) and who may have been asked to take part in a trial, you will have a useful perspective to offer about the information sheet and consent form. You may be asked to help write / edit / comment on these documents.

Helping develop on-going information about the trial for trial participants.

Trials sometimes develop websites / newsletters for trial participants. You may be asked to help develop or proof read the material for these to make sure that they are suitable and informative for patients / carers.

Promoting the trial

Whilst the trial is running and with the approval and guidance of the TMG you might decide that you would like to write a short piece about it for a voluntary organisation or patient group newsletter or website so that more people are aware of it and understand it. Or, again with the approval and guidance of the TMG you might want to give a short talk about the trial at a patient group meeting, locally or nationally. We understand that some people do not feel comfortable with speaking in public, so you do not need to feel obliged to do this. You may also be asked to help think of ways to make sure that patients, who might be eligible to take part in the trial, are aware that the trial exists in all trial sites.

Appendix 2—Examples of possible public contributor activities

Helping to tell people about the results of the trial

It is very important to get the message out about the results of a trial – not just to the clinicians and researchers but to patients and carers. You might want to help write or comment on the draft of a feedback sheet for people who took part in the trial. Or perhaps you might be interested in writing something about the trial for a patient group newsletter with the approval and guidance of the TMG. Some people decide that they would like to give a talk at a meeting or conference, but this differs from person to person

Trial participant experience

Some CTU trials look at what it is like for people who take part in the trial. Often a patient perspective can be really useful when thinking about what questions to ask trial participants about their experience.

Discussing the progress of the trial and any issues that arise

Over the course of a trial many issues arise which it is helpful and really important to have your public perspective on. Examples might be specific recruitment issues or problems with patients dropping out of the study early. Your expert view may help the group to consider alternative ways of addressing a recruitment or retention (early drop out) problem.

Study Logo

<Study name in full>

Appendix 3- Clinical Trial Timeline <Amend length of arrows according to trial timescales>

Stage in Clinical trial	Approximate timescales
Research funding agreed	6 months approx
Trial co-ordinator recruited & appointed	12 months approx
Study set up-period	
Recruitment of trial participants	<Insert approx. length of trial from initial recruitment to final follow up of all trial participants>
Trial participants in follow up	
Recruitment closes	
Follow up period closes	
Data entry completed	18 months approx
Data analysis completed	
Report and article writing	
Research findings published in an academic journal	

Note: The times in this timeline are approximate and may change, for example, if the trial needs to extend the period of recruitment

Appendix 4. The implications of being paid for involvement (*The following guidance is adapted from the INVOLVE document 'What you need to know about payments': http://www.invo.org.uk/wp-content/uploads/2011/06/INVOLVEpaymentdocument2011.pdf)*

Receiving payment of a fee for your involvement in research is likely to have implications for you whether you are currently employed, unemployed, receiving state benefits or retired. This is because the payment you receive will be treated as earnings. There are a number of ways in which receiving payment for involvement may affect your current financial situation:

If you are receiving state benefits:
Benefit rules on part-time earnings are complicated. The rules set limits on the amount you can be paid and the number of hours you can do. Different benefits have different earnings limits and these limits change from year to year. Nevertheless, it is important to remember that you can receive payment for involvement as long as it falls within the disregarded amount or earnings limit that applies to your benefits.

If the payments you receive exceed the earnings limit that applies to you or you have not informed Jobcentre Plus of your intention to become involved in research, you may be in danger of having your benefits reduced or withdrawn. It is your responsibility to get expert advice about this (for example, from your local Citizen's Advice Bureau or Jobcentre Plus) and to inform Jobcentre Plus if you are getting involved in research.

If you are receiving benefits because your health status or disabilities affect your ability to work, you will need to ensure that Jobcentre Plus understands that 'involvement' is different to employment. Jobcentre Plus has issued guidance to their staff explaining that involvement is not the same as work. In the text of a letter agreed with Jobcentre Plus policy team, it states: "Recruitment for involvement should not be confused with recruitment for employment" (INVOLVE Payment for Involvement, p.27)

If you are receiving retirement pension

Any earnings you receive in retirement counts as 'taxable income' along with income from your State Pension, personal or company (occupational) pensions and from certain taxable benefits. Bear in mind that any money you earn after State Pension age may affect income-related benefits such as Pension Credit, Housing Benefit and Council Tax Benefit. Staff at your pension centre can tell you how earnings may affect your Pension Credit. Your local authority can tell you how earnings may affect Housing Benefit and Council Tax Benefit.

Appendix 4. The implications of being paid for involvement (*The following guidance is adapted from the INVOLVE document 'What you need to know about payments': http://www.invo.org.uk/wp-content/uploads/2011/06/INVOLVEpaymentdocument2011.pdf)*

If you are liable for income tax and National Insurance

Payments made to you for involvement are usually subject to tax and National Insurance. We do not automatically deduct tax so you will need to notify HM Revenue and Customs of the payment if your annual income exceeds your personal income tax allowance at the end of the financial year. For further information and advice about this, you can contact your local HM Revenue and Customs office.

INVOLVE has information on how you can contact a free confidential benefits advice service specifically designed for public contributors in research– for further information see: http://www.invo.org.uk/resource-centre/benefits-advice-service/

Payment will be made directly into your bank account (account details will need to be provided at the point of making your claim).

Acknowledgements

The following documents were used in developing and preparing this remit document:

Becoming a consumer member of a Trial Management Group (TMG) in the Medical Research Council Clinical Trials Unit Cancer Group http://www.ctu.mrc.ac.uk/13706/13710/becoming_a_consumer_member_of_a_trial_management_group_in_the_mrc_ctu_cancer_group

National Institute for Health Research (NIHR) INVOLVE Briefing notes for researchers: public involvement in NHS, public health and social care research http://www.invo.org.uk/wp-content/uploads/2014/11/9938_INVOLVE_Briefing_Notes_WEB.pdf

NIHR INVOLVE - Public involvement in clinical trials: Supplement to the briefing notes for researchers. http://www.nihr.ac.uk/get-involved/INVOLVEpublicinvolvementinclinicaltrialsBriefingnotes2012.pdf

NIHR INVOLVE – What you need to know about payments. http://www.invo.org.uk/wp-content/uploads/2015/07/WhatYouNeedToKnowAboutPaymentUpdatedApril2015.pdf

Medicines for Children Research Network (MCRN) NIHR- Remit and Role Description for Parent/Carer Representatives on MCRN Clinical Studies Groups (CSGs).
