# Supplementary material for: A patient and public involvement (PPI) toolkit for meaningful and flexible involvement in clinical trials – a work in progress
Source: Res Involv Engagem. 2016 Apr 27;2:15. doi: 10.1186/s40900-016-0029-8 (PMC5611579; doi:10.1186/s40900-016-0029-8)
Supplement: Supplementary file 6 — PPI Remit TSC Public Contributor. (PDF 807 kb) [file 40900_2016_29_MOESM6_ESM.pdf]

<STUDY ACRONYM/LOGO>

<Study full name>

Trial Steering Committee (TSC) Public contributor - Remit & role description

This document outlines the background to both the <insert trial name> and the Trial Steering Committee and identifies the specific role and remit of patients, parents and carers who are members of this committee.

Information at a glance	
Your Role	Public contributor of the <trial name> Trial Steering Committee
Trial Duration	<insert trial start and end dates>
Duration of public contributor role	<Insert anticipated timescale of involvement required>
Frequency of involvement	<Insert anticipated frequency of involvement>
Location of involvement	Meetings will take place via teleconference <or describe alternative if meetings face to face, skype etc>
Expenses	<delete this row / amend as appropriate based on PPI funding in the trial> An honorarium will be paid for meeting attendance via teleconference
Key contact information<br><br><insert contact details of the TSC Chair>	<Name><br><br><telephone number><br><br><email address><br><br><address>

Contents **Page**

Background to the trial <#>

Background to the TSC <#>

The role of the public contributor <#>

Membership and key contacts <#>

Frequency and format of meetings <#>

Confidentiality <#>

Honorarium payment and expenses <#>

Evaluation and monitoring of the impact of patient and public involvement <#>

Appendix 1. Qualities of a public contributor <#>

Appendix 2. Trial Timelines <#>

Appendix 3. The implications of being paid for involvement <#>

Study Logo

<Study name in full>

What is the <Trial name> about?

<insert a very brief overview of the study, including who the trial is for and why it is being done (in plain English)>.

What is Patient and Public Involvement?

Public and Patient Involvement (PPI) and public contribution are often used interchangeably. In this document we use public contribution to refer to all such activities. In this context a public contributor refers to:

- *Children, with experience of, or interest in clinical research, health conditions and/or health settings who are interested in helping researchers design and run clinical trials*
- *Parents/carers/patients with experience of, or interest in clinical research, health conditions and/or health settings who are interested in helping researchers design and run clinical trials*

<delete depending on the trial>

What are trial oversight committees?

There are three groups that oversee the running of the trial (sometimes you will hear these three groups referred to collectively as the Trial Oversight Committees). These groups are:

- the Trial Steering Committee (TSC);
- the Trial Management Group (TMG) and
- the Independent Data and Safety Monitoring Committee (IDSMC).

The TSC, ISDMC and TMG have different roles but report to one another:

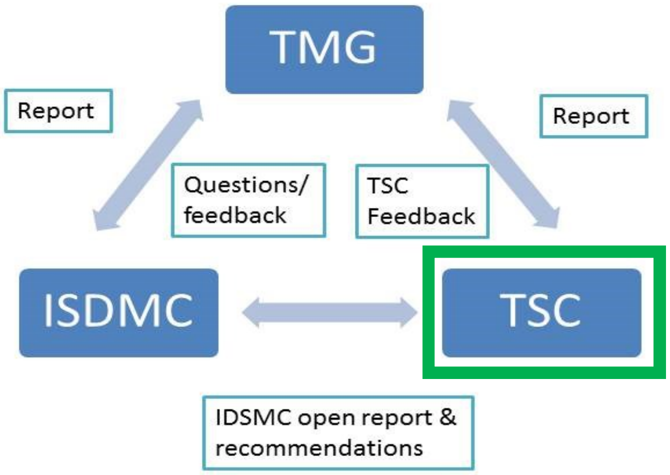

Both the TSC and the TMG usually have one or more members who are public contributors. It is important to involve people with experience of the condition to ensure the trial activities are considered from a patient perspective.

The qualities of a public contributor are in appendix 1.

Study Logo

<Study name in full>

What does the Trial Steering Committee do?

The TSC is the executive body that has overall oversight of the trial.

Membership of the TSC includes both independent members and some members of the trial team. To be independent a TSC member must:

- Not have been involved in designing the trial or applying for funding.
- Not be based at one of the sites involved.
- Not be involved in the day to day management of the trial.
- Not be part of the CTU coordinating the trial.
- Not work for the <insert institute name> who are acting as the trial Sponsor.

The TSC consider the trial from the perspective of the trial participants, trial team, the funder and the sponsor

Roles of the TSC include:

- To monitor the progress of the trial
- To monitor adherence to the protocol
- To consider recommendations from the Independent Data and Safety Monitoring Committee (IDSMC)
- To make recommendations to the Trial Management Group (TMG) .
- To make recommendations to the trial Sponsor.
- To consider new information as it becomes available

The full role of the TSC is described in the attached TSC terms of reference.

What does the Trial Management Group do?

The TMG is responsible for the day to day running of the clinical trial, including promotion and monitoring of the trial.

The TMG meet regularly, typically monthly, and review the ongoing progress and conduct of the trial including:

- Progress of trial and site opening
- Recruitment rate (actual versus predicted)
- Site issues
- Data quality and return rate
- Protocol amendments
- General trial issues

What does the Independent Data and Safety Monitoring Committee do?

The IDSMC is responsible for safeguarding the interests of <trial name> trial participants and for assessing both how safe and how effective the treatments being studied are. The IDSMC also review overall progress and conduct of the trial.

The members of the IDSMC are independent and not involved in the trial in any other way. Whilst the trial is running the IDSMC is the only committee that will see the trial data presented by treatment group. The TSC and TMG will only see data for the trial participants as a whole, not for individual treatment groups.

Study Logo

<Study name in full>

What is my role as Public Contributor member of the TSC?

The TSC is an independent multi-disciplinary committee. Each member, including public contributors, brings a different perspective, experience and knowledge to the trial team. Along with other members of the TSC you share the general role outlined in the TSC Terms of Reference (attached). As a public contributor we ask you to:

- Attend <amend as appropriate annual / bi-annual> TSC meetings (these may be teleconference or face-to-face) for the duration of the trial
- Provide a public perspective on TSC discussions
- Respond to written or verbal communications about the trial between meetings providing a public perspective
- Maintain the confidentiality of discussions and information relating to the TSC meetings and activities in accordance with the TSC confidentiality agreement (This is part of the TSC Terms of Reference).
- Report to the TSC any studies or information known to you which highlights patient related issues that may inform the work of the group.

The timeline for the <Trial Name> is described in Appendix 2. The TSC are usually in post until the publication of the main trial report.

How many meetings will I need to go to?

Make this trial specific. Example text:

<The TSC will meet annually for the duration of the trial>. However, the TSC may discuss the frequency of meetings and agree that more or less frequent meetings are needed.

Meetings typically last for about 1 ½ hours. An online “Doodle poll” is usually set up to agree meeting dates and times.

The <Trial name> Trial Coordinator will provide you with the TSC meeting dates, times and whether they will be a face to face meeting or by teleconference.

What happens if I can’t come to a meeting?

The TSC is made up of <X> independent members, including the public contributor For the TSC to be able to make decisions it is important that a minimum of <X> independent members attend.

The <Trial Name> will aim to find a date and time for the meeting that is suitable for everyone and it is important that you attend the meeting. We do understand that sometimes things change at the last minute and these can’t be avoided.

If you can’t attend a meeting but the rest of the TSC think your opinion is needed before making a decision a further meeting may be arranged or you might be asked to give your opinion by email.

Study Logo

<Study name in full>

Do I need to do anything in between meetings?

You may be asked to comment on documents by email between meetings but this will be minimal.

Whenever possible, if there are topics that you think should be included in the agenda for the TSC meeting please send these to the TSC chair or the trial coordinator 4 weeks before the TSC meeting date.

Confidentiality

All members of the TSC must abide by the confidentiality agreement set out in the <insert trial name> TSC Terms of Reference.

Who else is a member of the TSC?

The TSC is comprised of independent members and some members of the trial team. The independent members represent statistical, clinical. methodological and public contributor expertise.

A full list of all members of the TSC and contact details is given in <reference the template associated with the SOP here>.

Your main point of contact is the <NAME> who is the independent chairperson of the TSC.

Will I receive any payment or expenses?

<Delete / amend this section as appropriate based on funding within the grant, example text is provided>

Patient/public members of the TSC will be offered an honorarium payment for preparation, attendance and participation in the Trial Steering Committee. This will be based on current NIHR guidance.
and/or
You will receive reasonable travel expenses for attending face to face meetings.

You can find out what you can claim for on the next page. There are some things about receiving payment that you need to be aware of and we have summarised these in appendix 3.

.

Study Logo

<Study name in full>

What can I claim for? *amend this section as based on what PPI has been costed for, delete section if not appropriate.*

Public contributors are entitled to claim the following expenses for each agreed meeting / training they attend as a member of the TSC.

Travel to attend <delete as appropriate - face to face meetings / training >: (Remove this entire section if no funding for travel costs) Taxi, bus, train and underground fares and other incidentals will be reimbursed; taxis should only be used if cheaper transport is not practicable.

- Only standard /economy class transport costs can be claimed for.
- Attempts should be made when travelling to find the most economical route possible; this particularly applies to the purchase of rail tickets and air fares. Only standard / economy class rail and air travel can be claimed for.
- Car mileage is reimbursed at a rate of 45p per mile (up to the cost of a standard class rail ticket for the same journey).

Accommodation: <delete as appropriate in line with what has been costed for in terms of any accommodation costs, remove entire section if no costs for accommodation> will be offered when travelling for trial business involves <add in conditions of accommodation for example, length or journey, start time etc that will contribute to journey, start time etc that will contribute to the need for accommodation to be booked>. Accommodation should be booked via the trial coordinator prior to travel.

Childcare / carers: <Remove this entire section if no funding for registered childcare / carers>. All reasonable childcare / carer costs (with registered providers) will be covered up to <insert the details based on what has been costed for >

Subsistence: <delete as appropriate in line with what has been costed for in terms of attendance at any training / conferences / teleconferences>. (Remove this entire section if no costs allocated for subsistence in the trial). With receipts:

- Whilst travelling on < insert trial name> business involving an overnight stay or where travelling results in returning home after 8.00pm, employees may claim the cost of meals. For the avoidance of doubt we will not reimburse the cost of alcoholic drinks purchased while travelling on <insert trial name> business".
- FAQ -"Can I claim subsistence whilst away on <insert trial name> business but which does not include an overnight stay? YES, lunch (reasonable) including a non-alcoholic drink can be claimed, but not dinner unless after 8pm. Does not include snacks/unlimited teas/coffees/soft drinks throughout the day.

Study Logo

<Study name in full>

What you can claim for at a glance <Delete rows and columns as appropriate depending on PPI budget>

Meeting Type	Payment	Additional expenses
Attending a face to face meeting	<Insert amount e.g. £75 per meeting>	Cost of travel<br>Childcare/carers<br>(see section 10.4)
Attending a meeting by teleconference	<Insert amount eg. £40 per meeting>	Childcare/carers
Attending a training course as an observer * <insert as appropriate based on amount costed for - maximum of … conferences / training courses during the trial>	<insert amount eg. £30 per day>	The cost of travel, accommodation, subsistence and Childcare/carers<br>will be covered by the trial (see section 10.3)

***These activities must be agreed by the TMG / PPI co-ordinator first**

How can I make a claim for expenses? *amend /delete this section as based on what PPI has been costed for*

Travel can be booked in advance by the trial coordinator and is the preferred option that also means that you are not out of pocket.

If you find that you are no longer able to attend, where possible we would appreciate it if you could provide us with 24 hours' notice so that we can cancel any transport / accommodation (although we obviously understand that sometimes emergencies happen at the last minute and this is unavoidable).

All claims for travel, childcare and subsistence expenses must be supported by receipts. <delete as appropriate to what is funded in the trial>

A claim form and further information about what can be claimed and how to claim will be provided by the Trial Co-ordinator. Alternatively you can download a copy here: https://www.liv.ac.uk/intranet/media/intranet/humanresources/2014/myhr/formstore/payroll/Non-Staff,Expense,Form.pdf

The teleconference calls will be via a Freephone number when called from a landline number. These calls should also be free in the UK when called from a mobile (as of June 2015) but please check with your mobile 'phone provider.

Study Logo

<Study name in full>

Will I have to do anything else in my role as public contributor?

In addition to your role in the TSC we would also like you to be involved as a public contributor in the wider context of CTRC. This would involve:

- Completing public contributor evaluation forms about your experience .
- Ensuring any comments that you make in meetings that are taken on board for the trial are documented at each meeting (in both the minutes and the evaluation form).
- Contributing to consultation exercises carried out by CTRC in relation to public contributors (for example, we might want to see what you think about this remit document or our induction information).

Evaluation and monitoring of the impact of patient and public involvement

It is important for us, as coordinators of the trial, to evaluate the things that we do so that we can make changes and improve them.

After each TSC meeting we will ask you to complete an evaluation form.

Completing the evaluation form is voluntary but we would greatly value your feedback so that we can make sure we are giving you the right support.

We have tested the form and it should take you about 5 minutes to complete.

The main things we want to find out are:

- How you are enjoying your involvement
- What is being done well to involve you in the TSC
- What could be done better by the TSC
- Any suggestions you have

Study Logo

<Study name in full>

Who can I contact for more information?

If you would like more general information about being a public contributor on a research study please contact Heather Bagley (CTRC Patient and Public Involvement Co-ordinator – heather.bagley@liverpool.ac.uk).
Please note Heather works part-time and term-time only. Her working days are usually Monday – Thursday 9 – 2.30 although this occasionally varies

For information about the <trial name> contact:
<trial coordinator name, telephone number and email>

The National Institute for Health Research (NIHR) funds an organisation called INVOLVE which promotes active public participation in NHS, public health and social care research to improve the way that research is prioritised, commissioned, undertaken, communicated and used. INVOLVE have a range of resources which may be of value to public contributors - www.invo.org.uk

Other information you might find helpful

Appendix 1

Qualities of a public contributor

Appendix 2

Clinical Trial Timeline

Appendix 3

The implications of being paid for involvement

Study Logo

<Study name in full>

Appendix 1—Qualities of a public contributor

<Add or delete criteria as appropriate – for every project or panel there will be different criteria and needs>

Public contributors should have experience and knowledge of <insert the name of the condition>

- As a patient, or
- As a family member or carer of a patient

Essential criteria

- Have the time to be able to join bi-annual / annual TSC meetings
- Understanding of the issues relating to having <insert name of condition>
- Be able to maintain confidentiality (keeping information about the trial confidential, not discussing any information about the trial outside of the TSC)
- Ability to work effectively in a group situation
- Good communication skills with an ability to listen to others and constructively express a lay view beyond their own personal experience
- Have the time to attend meetings via teleconference / face to face meetings***<delete as appropriate for your trial, depending upon whether PPI was budgeted via teleconference or whether travel for face to face*** meetings was budgeted in>
- Have the time to comment on written information / emails between meetings

Desirable criteria

- Understanding of clinical trials or experience of taking part in a clinical trial
- Access to a computer, email and telephone. <This section may need adapting for certain populations where access to a computer and the internet could be a problem>

Study Logo

<Study name in full>

Appendix 2- Clinical Trial Timeline <Amend length of arrows according to trial timescales>

Stage in Clinical trial	Approximate timescales
Research funding agreed	6 months approx
Trial co-ordinator recruited & appointed<br>Study set up-period	12 months approx
Recruitment of trial participants<br>Trial participants in follow up<br>Recruitment closes<br>Follow up period closes	<Insert approx. length of trial from initial recruitment to final follow up of all trial participants>
Data entry completed<br>Data analysis completed<br>Report and article writing<br>Research findings published in an academic journal	18 months approx

Note: The times in this timeline are approximate and may change, for example, if the trial needs to extend the period of recruitment

Appendix 3. The implications of being paid for involvement (*The following guidance is adapted from the INVOLVE document 'What you need to know about payments': http://www.invo.org.uk/wp-content/uploads/2011/06/INVOLVEpaymentdocument2011.pdf)*

Receiving payment of a fee for your involvement in research is likely to have implications for you whether you are currently employed, unemployed, receiving state benefits or retired. This is because the payment you receive will be treated as earnings. There are a number of ways in which receiving payment for involvement may affect your current financial situation:

If you are receiving state benefits:
Benefit rules on part-time earnings are complicated. The rules set limits on the amount you can be paid and the number of hours you can do. Different benefits have different earnings limits and these limits change from year to year. Nevertheless, it is important to remember that you can receive payment for involvement as long as it falls within the disregarded amount or earnings limit that applies to your benefits.

If the payments you receive exceed the earnings limit that applies to you or you have not informed Jobcentre Plus of your intention to become involved in research, you may be in danger of having your benefits reduced or withdrawn. It is your responsibility to get expert advice about this (for example, from your local Citizen's Advice Bureau or Jobcentre Plus) and to inform Jobcentre Plus if you are getting involved in research.

If you are receiving benefits because your health status or disabilities affect your ability to work, you will need to ensure that Jobcentre Plus understands that 'involvement' is different to employment. Jobcentre Plus has issued guidance to their staff explaining that involvement is not the same as work. In the text of a letter agreed with Jobcentre Plus policy team, it states: "Recruitment for involvement should not be confused with recruitment for employment" (INVOLVE Payment for Involvement, p.27)

If you are receiving retirement pension

Any earnings you receive in retirement counts as 'taxable income' along with income from your State Pension, personal or company (occupational) pensions and from certain taxable benefits. Bear in mind that any money you earn after State Pension age may affect income-related benefits such as Pension Credit, Housing Benefit and Council Tax Benefit. Staff at your pension centre can tell you how earnings may affect your Pension Credit. Your local authority can tell you how earnings may affect Housing Benefit and Council Tax Benefit.

Appendix 3. The implications of being paid for involvement (*The following guidance is adapted from the INVOLVE document 'What you need to know about payments': http://www.invo.org.uk/wp-content/uploads/2011/06/INVOLVEpaymentdocument2011.pdf)*

If you are liable for income tax and National Insurance

Payments made to you for involvement are usually subject to tax and National Insurance. We do not automatically deduct tax so you will need to notify HM Revenue and Customs of the payment if your annual income exceeds your personal income tax allowance at the end of the financial year. For further information and advice about this, you can contact your local HM Revenue and Customs office.

INVOLVE has information on how you can contact a free confidential benefits advice service specifically designed for public contributors in research– for further information see: http://www.invo.org.uk/resource-centre/benefits-advice-service/

Payment will be made directly into your bank account (account details will need to be provided at the point of making your claim).

Acknowledgements

The following documents were used in developing and preparing this remit document:

Becoming a consumer member of a Trial Management Group (TMG) in the Medical Research Council Clinical Trials Unit Cancer Group http://www.ctu.mrc.ac.uk/13706/13710/becoming_a_consumer_member_of_a_trial_management_group_in_the_mrc_ctu_cancer_group

National Institute for Health Research (NIHR) INVOLVE Briefing notes for researchers: public involvement in NHS, public health and social care research http://www.invo.org.uk/wp-content/uploads/2014/11/9938_INVOLVE_Briefing_Notes_WEB.pdf

NIHR INVOLVE - Public involvement in clinical trials: Supplement to the briefing notes for researchers. http://www.nihr.ac.uk/get-involved/INVOLVEpublicinvolvementinclinicaltrialsBriefingnotes2012.pdf

NIHR INVOLVE – What you need to know about payments. http://www.invo.org.uk/wp-content/uploads/2015/07/WhatYouNeedToKnowAboutPaymentUpdatedApril2015.pdf

Medicines for Children Research Network (MCRN) NIHR- Remit and Role Description for Parent/Carer Representatives on MCRN Clinical Studies Groups (CSGs).
