# Supplementary material for: A patient and public involvement (PPI) toolkit for meaningful and flexible involvement in clinical trials – a work in progress
Source: Res Involv Engagem. 2016 Apr 27;2:15. doi: 10.1186/s40900-016-0029-8 (PMC5611579; doi:10.1186/s40900-016-0029-8)
Supplement: Supplementary file 7 — Participant Information Review Question Bank. (PDF 441 kb) [file 40900_2016_29_MOESM7_ESM.pdf]

Note blue text indicates text that should be amended on a trial by trial basis
**APPROACH**
Is it clear why you've been asked to take part?
Is it clear how long you will have to decide whether you want to take part or not?
**DETAILS OF INTERVENTION**
Do you understand why the study is being done?
Do you understand why this study needs to be done in this particular group of patients even though the medic
Do you understand what the medicine/device/intervention in the trial is/are?
Do you understand what is already known about the medicine/device/intervention?
Do you understand how the different trial medicines/devices/interventions work?
Is it clear that the <trial name> trial has <X> different medicines/devices/interventions?
Do you understand how and when you will receive the <insert name of medicine/device/intervention>?
Is it clear that the medicines/interventions/devices are only available by taking part in the trial?
**TRIAL AIM / PURPOSE**
Do you understand what the trial is trying to find out?
**FUNDING**
Is it clear who is funding the study?
**STANDARD CARE V TRIAL**
Do you understand the difference in the treatment that you will have if you take part in the study and if you d
Is it clear what will happen if a new treatment becomes available during the trial?
**BENEFITS**
Are the possible benefits of taking part clear?
Do you understand how you might help future patients by joining the study?
**SIDE EFFECTS**
Do you understand the risks of taking part in the study?
Do you understand the possible side effects of the treatment? < if there are long term and rare side effects th
Do you understand what you can do if you are harmed unexpectedly by taking part in the trial?
Is it clear that there *will/may/will not be* side effects from the *medicine/device/intervention* that you would r
Do you understand how the medicine/drug/intervention can affect your *ability to drive/use contraception/get*
**SCREENING**
Is it clear that before taking part you will have a <test/assessment> to make sure that it's ok for you to be in th
Is it clear how and when you will be told if you can take part or not?
Do you understand what will happen if your tests said you couldn’t take part?
**RANDOMISATION**
Do you understand what randomisation will mean in terms of the treatment you have?
Is it clear why randomisation is important?
Do you understand that you have an equal chance of getting treatment *A or B <need to add in details of what*
Do you understand how the treatment you get is decided?
**BLINDING**
Is it clear that being in a blinded study means that you won't know which treatment you are getting?
Is it clear why blinding is important?
Is it clear that in a medical emergency your doctor will be able to find out what treatment you have had so tha
**PARTICIPANT SCHEDULE**
Do you understand what will happen to you during the trial?
Are the tests/assessments that you will have made clear?
Do you understand what each of the different tests are?
Is it clear how many times you need to come to the hospital for an appointment?
Is it clear how long each appointment will last?

Do you know how long you will be in the study for in total?
Do you understand what will happen each time you come for an appointment?
Is it clear that the number or length of appointments is *different to/ the same* as standard treatment?
Is it clear where each appointment will take place?
Is it clear how many people will take part in the trial in total?
**EXPENSES**
Is it clear that you will receive expenses for your time/travel? *<only for trials where a patient receive payment*
Is it clear when and how expenses will be paid?
**NON PARTICIPATION**
Do you understand that that taking part is voluntary?
Is it clear that your care will not be affected if you decide not to take part in the trial?
**WITHDRAWAL**
Do you understand what to do if you don’t want to take part anymore?
Do you understand that you can choose to stop taking the medicine/using the device but can still contribute t
**DATA PROTECTION & CONFIDENTIALITY**
Do you understand who will have access to your records/information collected as part of the trial?
Do you understand how your confidentiality will be protected?
Is it clear that your *postcode and NHS number <or other identifiers>* will be collected?
Do you understand that you can choose for your *<NHS number or other optional identifier>* not to be collecte
**RESULTS / OUTCOME OF RESEARCH**
Is it clear when you will be able to find out the results of the trial as a whole?
Do you know how you can find out the results of the trial as a whole?
Is it clear how the results of the trial will be made available to a wider population?
Is it clear how will the results of the trial be used?
Is it clear if and when you will be made aware which *medicine/device/intervention you received? This is only a*
Is it clear if and when you will find out your own results?
**CONTACTS & ADVICE**
**Is it clear who to contact for more information?**
Is it clear who to contact if something is wrong during normal working hours *<insert time frame e.g. normal of*
Is it clear who to contact if something is wrong out of hours *<insert time frame e.g. out of hours, after 5pm etc*
Is it clear who to contact for more information or if you have any questions later on in the trial?
Is it clear what other sources of information are available about the trial? *<i.e. website, trial coordinator, DVD*
**GENERAL READABILITY**
Does the title of the information sheet make it clear what it's about?
Does the information sheet use headings to split sections up?
Does the information follow a logical order/flow well?
Is the writing concise (not rambling)?
Does the information sheet use everyday English without long, unfamiliar words?
Are any medical terms or procedures explained clearly?
Are there any terms, words or phrases that you find particularly daunting or scary in relation to taking part in t
Does the information sheet address you directly by using "you" and "we"?
Are the letters large and plain enough for you to read comfortably?
Is there enough space between the lines for you to read comfortably?
Are the lines of type too long to follow comfortably?
Are the sentences too long to follow clearly?
Are the diagrams (if any) clear?
Are the photographs/images(if any) appropriate?
Is colour used to make the document interesting?
Overall, do you feel that you could read, understand and act on the information given?
The development of these questions was informed by a published decision aid: Brown R

The development of these questions was informed by a published decision aid: Brown R, Shuk E, Butow, P et al. Identifying patient information needs about cancer clinical trials using a Question Prompt List, 2011. Patient Education & Counseling, 84, 1, pp. 69-77

cine/intervention has been used for others *<add details of the group that already use the medication/int*

on't?

en consider having a response option for each type i.e. long term, rare, etc>

ot get if you had standard treatment?

pregnant/take other medicines ?

e study?

the treatment options are and also whether its an equal chance or different ratio e.g. an equal chance of

t they can give you the right care?

, this is different to travel expenses etc>

o the trial by coming to follow up visits?

d?

pplicable to blinded trials, needs consideration of how protocol amendments could influence this aspect.

etc>

the trial?

ervention> ?

receiving surgery at 6 months or 12 months>?
