# Supplementary material for: A patient and public involvement (PPI) toolkit for meaningful and flexible involvement in clinical trials – a work in progress
Source: Res Involv Engagem. 2016 Apr 27;2:15. doi: 10.1186/s40900-016-0029-8 (PMC5611579; doi:10.1186/s40900-016-0029-8)
Supplement: Supplementary file 8 — Participant Information Review - Guidance for Public Contributors. (PDF 523 kb) [file 40900_2016_29_MOESM8_ESM.pdf]

<Trial Logo>

<Participant Information Sheet / Consent Form>

Information for reviewers

Introduction

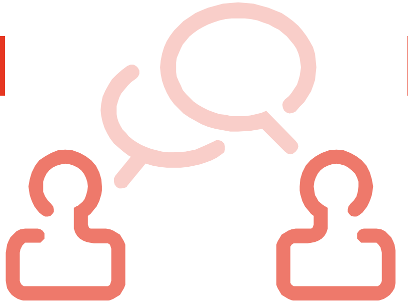

When a patient or member of the public is asked if they would like to take part in a clinical trial they are provided with two pieces of written information:

- A participant information sheet
- A consent / assent form (assent is a child's agreement to take part)

It is very important that those invited to take part in a trial or study understand fully what is involved before they make their decision.

For this reason the Clinical Trials Research Centre is very keen to ask patients / the public to read and comment on our information before it is used in a clinical trial.

Your comments and suggestions will help us to improve the information that we give to potential trial participants.

What we would like you to do

We would like you to read the information on page 2 of this document which explains the *<trial name>* and the situation that potential participants might be in when they are asked if they would like to take part in the trial.

Keeping this information in mind we would then like you to do the following:

- Read through the information sheet and consent form *<insert alternative document type if being reviewed>*

- Add any comments, suggestions or revisions you might have. You can do this in the way that you prefer. For example:
  - ⇒ Write notes on printed copies of the documents
  - ⇒ Make changes directly in the Word document using a different colour text to mark your changes or by using the "track changes" function
  - ⇒ Make your own notes in a notebook/document.

Optional

We've also put together a list of some questions that you might like to think about as you read the information sheet.

These are still a work in progress but if you would like to see a use them they are on pages 3 - 4 of this Information for Reviewers document.

How should I send my feedback?

You can send your feedback in the following ways:

By email to <Name> < trial email>

Include any documents that you have made comments on for example, an edited word document or a scan of your notes on a printed copy

By post

Include any notes you've made, making sure you've written your name on them, and return using the to:

Name
Address 1 ,Address 2, Address 3, Postcode

By phone

If you would prefer you can also give your feedback over the phone by contacting <Trial Coordinator> on <telephone number>

When do I need to send my feedback by?

We would welcome your comments by:

<day-month-year>

If you would like to give your feedback by telephone then we can arrange a suitable time to contact you.

Documents you'll need

- This information for reviewers
- A copy if the <trial name> information sheet and consent form (version number and date) *<or list which documents>*

Documents that you might need

- A version of the <trial name> information sheet and consent form in Microsoft Word so that changes can be tracked
- A version of the formatted *<document name>* has also been included so that you can see what the finished product will look like. This is as a guide only and we will update it when we have had feedback on the content.

UNIVERSITY OF LIVERPOOL

<Trial Logo>

CTRC Clinical Trials Research Centre

Information for you about the <Trial Name>

<In this section we are trying to help the person reading the information to imagine the situation of a potential participant who might be approached to take part in the study. They need to know:

- Whether the person would be a healthy participant (not a patient) eg. Phase 1 studies or vaccine studies or a patient.
- What age and gender the potential trial participant would be
- Where the participant might be in their clinical journey (at the point of diagnosis / several years into treatment)
- What setting the potential participant would receive this information in (eg. on an intensive care unit, in an out-patient clinic, through the post)
- Whether there are any other special considerations e.g. if the participant would be receiving the information in a deferred consent situation (explain this in plain English to the person reading the information)

Provide this information in a simple format using bullet points if possible

You should also consider any flow charts or diagrams that might help to explain the trial

Ensure plain language is used throughout >

Some questions……..

We’ve come up with some questions that you might like to think about when your read the information sheet.

You don’t have to use these if you don’t want to.

Is it clear why you've been asked to take part?

Do you understand what the trial is trying to find out?

Is it clear what the medicine/device/ intervention being used in the study is?

Is it clear how long you will have to decide whether you want to take part or not?

Is it clear what is already know about the different medicines/devices/ interventions in the study

Is it clear that before taking part you will have <test/ assessment> to make sure that its ok for you to be in the study?

Is it clear how and when you will be told if you can take part or not?

Are the possible benefits of taking part clear?

Do you understand how you might help future patients by joining the study?

Do you understand the possible side effects of the treatment?

Do you understand the risks of taking part in the study?

Is it clear that there will/may be side effects from the medicine/device/ intervention that you would not get if you had standard care?

Do you understand how the medicine/ drug/intervention can affect your ability to drive/use contraception/get pregnant/take other medicines ?

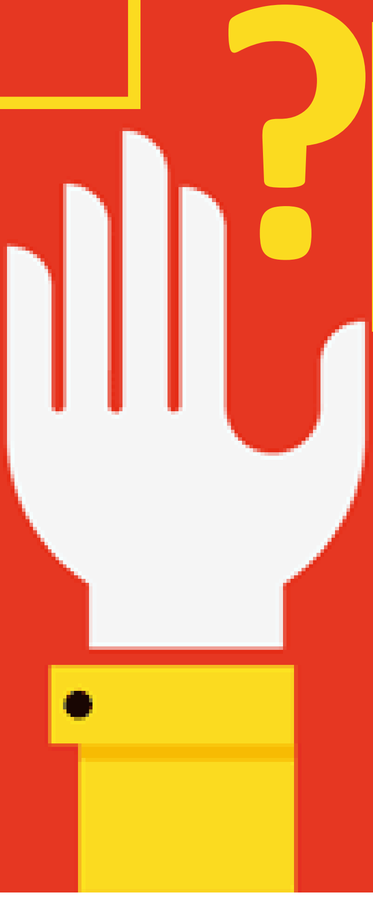

Do you understand the difference in the treatment that you will have if you take part in the study and if you don’t?

Do you understand how and when you will recive the <insert name of medicine/device/ intervention>

Is it clear what will happen if a new treatment becomes available during the study?

Some more questions........

Do you understand what randomisation will mean in terms of your treatment?

Do you understance that you have an equal chance of getting either treatment? <amend depending on randomisation/treatments>

Is it clear that the medicines/ interventions/devices are only available by taking part in the trial?

Do you understand what will happen to you during the trial?

Is it clear how many times you need to come to the hospital for an appointment?

Is it clear how long each appointment will last?

Do you know how long you will be in the study in total?

Do you understand what you can do if you are harmed unexpectedly by taking part in the trial?

Are contact details of the study team easy to find?

Is it clear who is funding the study?

Is it clear that being in a blinded study means that you won't know which treatment you are getting?

Is it clear why blinding is important?

Is it clear that in a medical emergency your doctor will be able to find out what treatment you have had so that they can give you the right care?

Do you understand what will happen each time you come for an appointment?

Are the tests/assessments that you will have clear?

Do you understand what each of the different tests are?

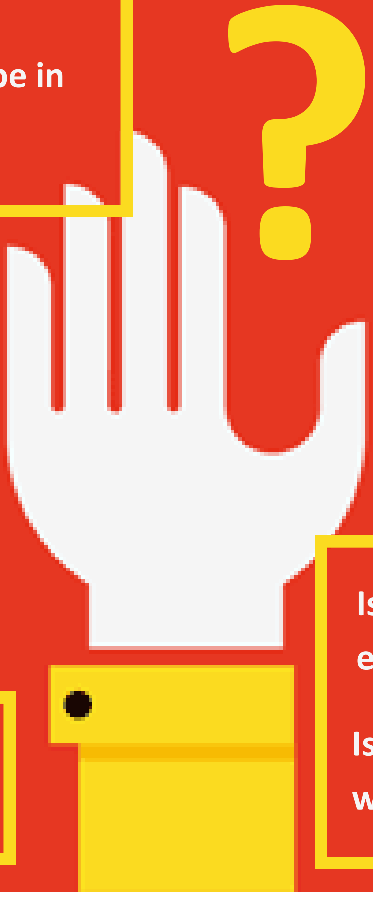

Do you understand that you can stop taking part at any time without giving a reason?

Do you understand what to do if you decide that you don't want to take part anymore?

Is it clear that you will receive expenses for your time/travel?

Is it clear when and how expenses will be paid?
